# Clinical and Immunological Factors That Distinguish COVID-19 From Pandemic Influenza A(H1N1)

**DOI:** 10.3389/fimmu.2021.593595

**Published:** 2021-04-21

**Authors:** José Alberto Choreño-Parra, Luis Armando Jiménez-Álvarez, Alfredo Cruz-Lagunas, Tatiana Sofía Rodríguez-Reyna, Gustavo Ramírez-Martínez, Montserrat Sandoval-Vega, Diana Lizzeth Hernández-García, Eduardo M. Choreño-Parra, Yalbi I. Balderas-Martínez, Mariana Esther Martinez-Sánchez, Eduardo Márquez-García, Edda Sciutto, José Moreno-Rodríguez, José Omar Barreto-Rodríguez, Hazel Vázquez-Rojas, Gustavo Iván Centeno-Sáenz, Néstor Alvarado-Peña, Citlaltepetl Salinas-Lara, Carlos Sánchez-Garibay, David Galeana-Cadena, Gabriela Hernández, Criselda Mendoza-Milla, Andrea Domínguez, Julio Granados, Lula Mena-Hernández, Luis Ángel Pérez-Buenfil, Guillermo Domínguez-Cheritt, Carlos Cabello-Gutiérrez, Cesar Luna-Rivero, Jorge Salas-Hernández, Patricio Santillán-Doherty, Justino Regalado, Angélica Hernández-Martínez, Lorena Orozco, Federico Ávila-Moreno, Ethel A. García-Latorre, Carmen M. Hernández-Cárdenas, Shabaana A. Khader, Albert Zlotnik, Joaquín Zúñiga

**Affiliations:** ^1^ Escuela Nacional de Ciencias Biológicas, Instituto Politécnico Nacional, Mexico City, Mexico; ^2^ Laboratory of Immunobiology and Genetics, Instituto Nacional de Enfermedades Respiratorias Ismael Cosío Villegas, Mexico City, Mexico; ^3^ Department of Immunology and Rheumatology, Instituto Nacional de Ciencias Médicas y Nutrición Salvador Zubirán, Mexico City, Mexico; ^4^ Facultad de Estudios Superiores Iztacala, Universidad Nacional Autónoma de México, Mexico City, Mexico; ^5^ Intensive Care Unit, Instituto Nacional de Enfermedades Respiratorias Ismael Cosío Villegas, Mexico City, Mexico; ^6^ Posgrado en Ciencias Biológicas, Universidad Nacional Autónoma de México, Mexico City, Mexico; ^7^ Department of Immunology, Instituto de Investigaciones Biomédicas, Universidad Nacional Autónoma de México, Mexico City, Mexico; ^8^ Direccion de Enseñanza e Investigación, Hospital Juárez de Mexico, Mexico City, Mexico; ^9^ Subdirección de Medicina, Instituto Nacional de Enfermedades Respiratorias Ismael Cosío Villegas, Mexico City, Mexico; ^10^ Coordinación de Infectología y Microbiología, Instituto Nacional de Enfermedades Respiratorias Ismael Cosío Villegas, Mexico City, Mexico; ^11^ Departamento de Neuropatología, Instituto Nacional de Neurología y Neurocirugía “Manuel Velasco Suarez”, Mexico City, Mexico; ^12^ Departamento de Fibrosis Pulmonar, Instituto Nacional de Enfermedades Respiratorias Ismael Cosío Villegas, Mexico City, Mexico; ^13^ Tecnologico de Monterrey, Escuela de Medicina y Ciencias de la Salud, Mexico City, Mexico; ^14^ Department of Transplantation, Instituto Nacional de Ciencias Médicas y Nutrición Salvador Zubirán, Mexico City, Mexico; ^15^ Department of Dermatology, Instituto Nacional de Ciencias Médicas y Nutrición Salvador Zubirán, Mexico City, Mexico; ^16^ Department of Education, Instituto Nacional de Ciencias Médicas y Nutrición Salvador Zubirán, Mexico City, Mexico; ^17^ Critical Care Unit, Instituto Nacional de Ciencias Médicas y Nutrición Salvador Zubirán, Mexico City, Mexico; ^18^ Department of Virology, Instituto Nacional de Enfermedades Respiratorias Ismael Cosío Villegas, Mexico City, Mexico; ^19^ Department of Pathology, Instituto Nacional de Enfermedades Respiratorias Ismael Cosío Villegas, Mexico City, Mexico; ^20^ General Direction, Instituto Nacional de Enfermedades Respiratorias Ismael Cosío Villegas, Mexico City, Mexico; ^21^ Department of Medical Direction, Instituto Nacional de Enfermedades Respiratorias Ismael Cosío Villegas, Mexico City, Mexico; ^22^ Laboratorio Inmunogenómica y Enfermedades Metabólicas, Instituto Nacional de Medicina Genómica, Mexico City, Mexico; ^23^ Biomedicine Research Unit (UBIMED), Lung Diseases and Cancer Epigenomics Laboratory, Facultad de Estudios Superiores (FES) Iztacala, Universidad Nacional Autónoma de México (UNAM), Tlalnepantla de Baz, Mexico; ^24^ Department of Molecular Microbiology, Washington University School of Medicine in St Louis, St Louis, MO, United States; ^25^ Department of Physiology & Biophysics School of Medicine, Institute for Immunology, University of California, Irvine, CA, United States

**Keywords:** SARS-CoV-2, COVID-19, Influenza A(H1N1) pdm09, pandemic influenza, acute respiratory distress syndrome

## Abstract

The severe acute respiratory syndrome coronavirus 2 (SARS-CoV-2), the causative agent of coronavirus disease 2019 (COVID-19), is a global health threat with the potential to cause severe disease manifestations in the lungs. Although COVID-19 has been extensively characterized clinically, the factors distinguishing SARS-CoV-2 from other respiratory viruses are unknown. Here, we compared the clinical, histopathological, and immunological characteristics of patients with COVID-19 and pandemic influenza A(H1N1). We observed a higher frequency of respiratory symptoms, increased tissue injury markers, and a histological pattern of alveolar pneumonia in pandemic influenza A(H1N1) patients. Conversely, dry cough, gastrointestinal symptoms and interstitial lung pathology were observed in COVID-19 cases. Pandemic influenza A(H1N1) was characterized by higher levels of IL-1RA, TNF-α, CCL3, G-CSF, APRIL, sTNF-R1, sTNF-R2, sCD30, and sCD163. Meanwhile, COVID-19 displayed an immune profile distinguished by increased Th1 (IL-12, IFN-γ) and Th2 (IL-4, IL-5, IL-10, IL-13) cytokine levels, along with IL-1β, IL-6, CCL11, VEGF, TWEAK, TSLP, MMP-1, and MMP-3. Our data suggest that SARS-CoV-2 induces a dysbalanced polyfunctional inflammatory response that is different from the immune response against pandemic influenza A(H1N1). Furthermore, we demonstrated the diagnostic potential of some clinical and immune factors to differentiate both diseases. These findings might be relevant for the ongoing and future influenza seasons in the Northern Hemisphere, which are historically unique due to their convergence with the COVID-19 pandemic.

## Introduction

The novel SARS-CoV-2 has submerged the world into a public health crisis of unprecedented features. With more than 113.4 million infected people and 2.5 million deaths, SARS-CoV-2 continues spreading worldwide (1). Although other emerging pathogens have caused similar outbreaks in the past, the pandemic influenza A(H1N1) pdm09 virus is the immediate antecedent reference for the global spread of a new zoonotic respiratory pathogen. This virus emerged in Mexico in 2009, causing approximately 151,700-575,400 deaths worldwide during the first year after its appearance (2–4). Ever since, the influenza A(H1N1) pdm09 virus has continued circulating globally, acquiring a seasonal transmission pattern (5). Notably, the emergence of SARS-CoV-2 in December of 2019 (6–8), occurred when several countries were at the peak of the flu season. This hampered differentiating COVID-19 and influenza during the early days of the current pandemic. With improved understanding of the clinical characteristics and pathobiology of COVID-19 (9–12), the overall identification of positive cases drastically improved.

Despite this, only a few comparisons of the characteristics of COVID-19 and influenza have been conducted (13–16). This is crucial as both entities are converging at several regions of the Northern hemisphere. In this context, the accurate identification of the causative pathogen has important therapeutic implications, including the selection of adequate antiviral drugs. A better understanding of the host factors implicated in protective vs. pathogenic immunity against SARS-CoV-2 is also crucial to guide immunotherapeutic interventions for patients in critical conditions. Unfortunately, what we currently comprehend about the immunopathology of severe COVID-19 is a paradox: the adaptive response is overactive but unable to control the virus. In fact, patients with COVID-19 display a pro-inflammatory (IL-1β, IL-6, IL-7, IL-8, IL-9, FGF, G-CSF, GM-CSF, IFN-ɣ, CXCL10, CCL2, CCL3, CCL4, PDGF, TNFα, and VEGF) and regulatory cytokine profile (IL-10 and TGFβ; cytokine storm) (17, 18). Interestingly, unlike other cytokine storm syndromes, the polyfunctional immune activation of COVID-19 is accompanied by lymphopenia, reduced T cell numbers, and strong infiltration of immune cells into the lung (19–21). Thus, the lung damage associated with COVID-19 may be caused both by the virus and hyperinflammation.

Comparing the immune profiles of COVID-19 with other respiratory pathogens may dissipate prevailing controversies about the immunopathology of SARS-CoV-2 infection. For this reason, here we evaluated clinical and immunological factors distinguishing critically ill COVID-19 and pandemic influenza A(H1N1) patients. We also compared histopathological changes and expression of immune markers in the lungs of patients with both diseases. Our results reveal crucial differences in the clinical characteristics of the two infections. Furthermore, our analyses clearly show that the human immune response elicited after SARS-CoV-2 is completely different from the immune responses against the influenza A(H1N1) pdm09 virus. Our study may support the use of some of these distinctive traits to differentiate COVID-19 from pandemic influenza A(H1N1) reliably.

## Materials and Methods

### Participants

We conducted a prospective cohort study in patients with an acute respiratory illness that attended the emergency department of the Instituto Nacional de Ciencias Médicas y Nutrición Salvador Zubirán (INCMNSZ), and the Instituto Nacional de Enfermedades Respiratorias Ismael Cosío Villegas (INER) in Mexico City. Individuals with laboratory-confirmed COVID-19 requiring hospital admission were eligible. Detection of SARS-CoV-2 was performed by real-time polymerase chain reaction (RT-PCR) in swab samples, bronchial aspirates (BA), or bronchoalveolar lavage (BAL) specimens, as previously described (22). Briefly, viral RNA was extracted from clinical samples with the MagNA Pure 96 system (Roche, Penzberg, Germany). The RT-PCR reactions were performed in a total volume of 25μL, containing 5μL of RNA, 12.5μL of 2 × reaction buffer provided with the Superscript III one-step RT-PCR system with Platinum Taq Polymerase (Invitrogen, Darmstadt, Germany; containing 0.4 mM of each deoxyribose triphosphates (dNTP) and 3.2 mM magnesium sulfate), 1μL of reverse transcriptase/Taq mixture from the kit, 0.4μL of a 50 mM magnesium sulfate solution (Invitrogen), and 1μg of nonacetylated bovine serum albumin (Roche). Primer and probe sequences, as well as optimized concentrations, are shown in **Supplemental Table 1**. All oligonucleotides were synthesized and provided by Tib-Molbiol (Berlin, Germany). Thermal cycling was performed at 55°C for 10 min for reverse transcription, followed by 95°C for 3 min and then 45 cycles of 95°C for 15 s, 58°C for 30 s.

Individuals with COVID-19 were further categorized into two groups: a) moderate COVID-19 group (n=10), that included patients with respiratory symptoms that did not require mechanical ventilation (MV); and b) severe COVID-19 group (n=24), consisting of patients requiring invasive MV and admission to the intensive care unit (ICU). Our comparative cohort included patients with influenza-like illness (ILI) that attended to the INER in Mexico City during the immediately preceding 2019/2020 flu season. Individuals with confirmed influenza A(H1N1) pdm09 virus infection that progressed to acute respiratory distress syndrome (ARDS), requiring MV and admission to the ICU were included. ILI was defined as an acute respiratory illness with a measured temperature of ≥ 38°C and cough, with onset within the past ten days. These subjects were first screened for influenza A virus infection using the Fuji dri-chem immuno AG cartridge FluAB kit (Fujifilm Corp, Tokyo, Japan) rapid influenza diagnostic test (RIDT) in fresh respiratory swab specimens. In positive cases, further molecular characterization of the causative influenza A virus subtype was assessed by RT-PCR. All influenza cases enrolled in the study were infected with the pandemic influenza A(H1N1) pdm09 virus. None of the participants had human immunodeficiency virus (HIV) infection.

### Data Retrieval

Microsoft Excel (MS Excel 365) was used for data collection. Clinical and demographic data were retrieved from all participants´ medical records. These data included age, gender, anthropometrics, comorbidities, symptoms, triage vital signs, the severity of illness scores at admission [Sequential Organ Failure Assessment (SOFA), and Acute Physiology And Chronic Health Evaluation II (APACHE II)], and initial laboratory tests. Initial laboratory tests were defined as the first test results available (typically within 24 hours of admission) and included white blood cell counts, liver and kidney function, gasometric parameters at admission, and other tissue-injury biomarkers.

### Cytokine Determinations

Peripheral blood samples were obtained from all participants at hospital admission. Serum levels of different cytokines, chemokines, growth factors, and other immune mediators were determined by Luminex assays using the Luminex platform Bio-Plex Multiplex 200 (Bio-Rad Laboratories, Inc., Hercules, CA, USA). Serum samples from 13 healthy donors were used as controls. The immune mediators that were quantified are listed as follows: IFN-α, interferon-alpha, IFN-β; interferon-beta; IFN-γ, interferon-gamma; TNF-α, tumor necrosis factor-alpha; IL-1β, interleukin 1beta; IL-1RA, interleukin 1 receptor antagonist; IL-2, interleukin 2; IL-4, interleukin 4; IL-5, interleukin 5; IL-6, interleukin 6; IL-7, interleukin 7; IL-8, interleukin 8; IL-9, interleukin 9; IL-10, interleukin 10; IL-12 (p40), interleukin 12 p40 subunit; IL-12p70, interleukin 12 p70 subunit; IL-13, interleukin 13; IL-15, interleukin 15; IL-17A, interleukin 17A; IL-26, interleukin 26; IL-32, interleukin 32; CXCL10, C-X-C motif chemokine ligand 10, CCL2, C-C motif chemokine ligand 2; CCL3, C-C motif chemokine ligand 3; CCL4, C-C motif chemokine ligand 4; CCL5, C-C motif chemokine ligand 5; CCL11, C-C motif chemokine ligand 11; G-CSF, granulocyte colony-stimulating factor; bFGF, basic fibroblast growth factor; PDGF-BB, platelet-derived growth factor bb; VEGF, vascular endothelial growth factor; APRIL/TNFSF13, A proliferation-inducing ligand/tumor necrosis factor ligand superfamily member 13; BAFF/TNFSF13B, B-cell activating factor/tumor necrosis factor ligand superfamily member 13B; sCD30/TNFRSF8, soluble CD30/tumor necrosis factor ligand superfamily member 8; sCD163, soluble CD163; chitinase 3/like1; gp130/sIL-6Rβ, glycoprotein of 130 kDa/soluble IL-6 receptor beta; sIL-6Rα, soluble IL-6 receptor alpha; MMP-1, matrix metalloprotease 1; MMP-2, matrix metalloprotease 2; MMP-3, matrix metalloprotease 3; osteocalcin; ostepontin; pentraxin-3; sTNF-R1, soluble tumor necrosis factor receptor 1; sTNF-R2, soluble tumor necrosis factor receptor 2; TSLP, thymic stromal lymphopoietin; TWEAK/TNFSF12, tumor necrosis factor-like weak inducer of apoptosis/tumor necrosis factor ligand superfamily member 12.

### Histopathological Analysis

Formalin-fixed and paraffin-embedded lung autopsy specimens from patients who died of pandemic influenza A(H1N1) or COVID-19 (N=2 patients per group) were obtained from the Pathology Department of the INER. Sections of 3-5μm were processed for hematoxylin-eosin (H&E) staining for histopathological analysis. For immunohistochemistry (IHQ), lung sections were mounted on silane-covered slides, deparaffinized in xylenes, and hydrated with a series of graded alcohol-to-water dilutions. The endogenous peroxidase was blocked with 3% hydrogen peroxide for 30 minutes. Sections were incubated overnight at room temperature with optimal dilutions (1:100) of the following antibodies: anti-IFN-γ (Anti-Interferon gamma antibody, ab9657, Abcam, UK), anti-IL-1β (IL-1β Antibody (H-153): sc-7884, Santa Cruz Biotechnology Inc., Santa Cruz, CA), anti-IL-4 (IL-4 Antibody (OX81): sc-53084, Santa Cruz Biotechnology Inc., Santa Cruz, CA), and anti-IL-17A (Anti-IL-17 antibody (ab91649), Abcam, UK). Secondary biotinylated antibodies labeled with peroxidase were added, and those attached were revealed with diaminobenzidine (DAB) for 5 minutes (MACH 1 Universal HRP-Polymer Detection Kit, Biocare Medical, LLC). Slides were counter-stained with hematoxylin.

### Statistical Analysis

Descriptive statistics were used to characterize the study population clinically. Frequencies and proportions were calculated for categorical data. Means, medians, standard deviations (SD), interquartile ranges (IQR), and 95% confidence intervals were used for continuous variables. Differences between groups were assessed by the Fisher exact, Chi-square test, Mann-Whitney U test, or Kruskal-Wallis test with *post hoc* Dunn´s test, as appropriate. Multiple linear regression analyses using Spearman rank correlation coefficients were used to determine correlations between continuous variables. ROC curves were constructed to estimate the diagnostic utility of different variables to differentiate between participant groups in terms of their area under the curve (AUC). The prognostic value of the different clinical and immunological parameters expressed in terms of odds ratio (OR) values for adverse outcomes (intubation, death) was estimated using binomial logistic regression analyses.

Principal component analyses (PCAs) were conducted to analyze how the study participants clustered together according to the interplay between their clinical and immunological characteristics. Furthermore, a Linear Discriminant Analysis (LDA) without and with “leave-one-out” type cross-validation was performed to assess whether the linear combination of different variables allowed differentiating individuals according to their diagnosis. The variables included were AST, ALT, LDH, ALP, procalcitonin, SOFA, IL-1β, IL-1RA, IL-2, IL-4, IL-5, IL-7, IL-12, IL-13, IL- 17A, TNF-α, CCL3, CCL11, G-CSF, and VEGF. A Wilks ‘Lambda test was performed to evaluate the discriminatory power of each variable in the LDA. Variables were transformed to log10 to meet the LDA assumptions and were scaled to prevent the scale of each variable from influencing the analysis results. Individuals with missing data were omitted from PCA and LDA analyses. All analyses were conducted using GraphPad Prism 8 (La Jolla, CA), R Statistical Software (Foundation for Statistical Computing, Vienna, Austria) packages Factoextra and MASS, and Python packages pandas v0.23.4 and seaborn v0.10.1. Specific analysis tests are also mentioned in figure legends. P values ≤0.05 were considered as significant: *p ≤ 0.05, **p ≤ 0.01, ***p ≤ 0.001, ****p ≤ 0.0001.

### Study Approval

The Institutional Review Boards of the INCMNSZ (approval number: 3349) and the INER (approval number: B28-16 and B09-20) in Mexico City approved the study. All participants or their legal guardians provided written informed consent in accordance with the Declaration of Helsinki for Human Research. Clinical samples were managed according to the Mexican Constitution law NOM-012-SSA3-2012, which establishes the criteria for the execution of clinical investigations in humans.

## Results

### Participant Characteristics

The main demographic characteristics of enrolled patients were similar (**Table 1**), although the proportion of males tended to be higher in both groups of COVID-19 subjects, as reported before (9, 11, 12, 17, 23, 24). Obesity was more frequent in pandemic influenza A(H1N1) patients, whereas other comorbidities (diabetes, systemic arterial hypertension (SAH), chronic obstructive pulmonary disease (COPD), and obstructive sleep apnea syndrome (OSA)) were equally distributed across groups. Fever was the most frequent symptom among all participants, followed by cough, fatigue, myalgia, arthralgia, and headache. Dyspnea occurred in 10% of patients with moderate COVID-19 and in ~80% of individuals with severe COVID-19 and pandemic influenza A(H1N1). Rhinorrhea, sore throat, thoracic pain, and sputum production were more common during pandemic influenza A(H1N1), whereas dry cough, diarrhea, and vomit were more frequent among COVID-19 patients. This finding suggests that some symptoms could differentiate these infectious entities. We performed a logistic regression analysis with the symptoms reported by pandemic influenza A(H1N1) and COVID-19 patients at hospital admission. Fever and rhinorrhea were associated with pandemic influenza A(H1N1), whereas dry cough predicted COVID-19 (**Supplemental Figure 1** and **Supplemental Table 2**). Sore throat and thoracic pain were marginally associated with pandemic influenza A(H1N1) but did not reach statistical significance. Similarly, gastrointestinal symptoms exhibited higher, but not significant odds ratio (OR) values for COVID-19 (**Supplemental Figure 1** and **Supplemental Table 2**). Overall, patients in the moderate COVID-19 group attended earlier after symptoms onset than individuals with severe pandemic influenza A(H1N1) and COVID-19 (**Table 1**).

**Table 1:** Clinical characteristics of patients with COVID-19 and pandemic influenza.

Characteristic	Influenza A(H1N1) pdm09A N = 23	*p*-value A vs. B	Moderate COVID-19BN = 10	p-value B vs. C	Severe COVID-19CN = 24	p-value A vs. C
**Age (years), median (range)**	49 (29 - 77)	0.2385	34.5 (28 – 71)	0.1706	52 (30 – 73)	>0.9999
**Gender** **Males** **Females**	14 (60.86) 9 (39.13)	0.7098	7 (70) 3 (30)	0.5659	19 (79.16) 5 (20.83)	0.1703
**BMI**	33.6 (29.6 - 42.4)	0.0004	25.3 (22.5 – 29.3)	0.1164	29.6 (25.3 – 33.4)	0.0592
**Relevant co-morbidities** **Smoking** **Biomass exposure** **Diabetes** **SAH** **OSA** **COPD** **Cancer**	8 (34.78) 5 (21.73) 5 (21.73) 6 (26.08) 2 (8.69) 2 (8.69) 0 (0)	0.0715 0.2911 >0.9999 >0.9999 >0.9999 >0.9999 0.0852	0 (0) 0 (0) 2 (20) 2 (20) 0 (0) 1 (10) 2 (20)	0.0720 0.2958 >0.9999 >0.9999 1 0.2941 0.0802	8 (33.33) 4 (16.66) 6 (25) 4 (16.66) 0 (0) 0 (0) 0 (0)	0.9165 0.7238 0.7918 0.4936 0.2340 0.2340 1
**Clinical findings at onset** **Fever** **Myalgia** **Arthralgia** **Headache** **Dyspnea** **Nasal congestion** **Rhinorrhea** **Sore throat** **Thoracic pain** **Cough** **Sputum** **Dry cough** **Fatigue** **Diarrhea** **Nausea** **Vomit**	21 (91.3) 17 (73.91) 17 (73.91) 11 (47.82) 18 (78.26) 3 (13.04) 11 (47.82) 8 (34.78) 4 (17.39) 19 (82.6) 11 (47.82) 8 (34.78) 19 (82.6) 2 (8.69) 2 (8.69) 0 (0)	0.3605 0.7077 0.4438 0.9086 0.0004 0.5363 0.0129 0.0321 0.2890 0.4155 0.0129 0.0619 >0.9999 0.1493 0.1493 0.0220	8 (80) 8 (80) 6 (60) 5 (50) 1 (10) 0 (0) 0 (0) 0 (0) 0 (0) 7 (70) 0 (0) 7 (70) 8 (80) 3 (30) 3 (30) 3 (30)	0.7541 0.5809 0.7109 0.8245 0.0002 >0.9999 0.1478 0.1478 1 0.2226 0.2958 0.9612 0.9563 0.3943 0.1380 0.3284	18 (75) 17 (70.83) 16 (66.66) 11 (45.83) 19 (79.16) 2 (8.33) 6 (25) 6 (25) 0 (0) 21 (87.5) 4 (16.66) 17 (70.83) 19 (79.16) 4 (16.66) 2 (8.33) 3 (12.5)	0.1371 0.8135 0.5871 0.8911 0.9395 0.6662 0.1035 0.4635 0.0496 0.6378 0.0220 0.0133 0.7643 0.6662 >0.9999 0.0797
**Illness onset - hospital admission (days)**	7 (4 - 8.5)	0.0583	3 (0 – 5.7)	0.0158	6 (5 – 13.2)	>0.9999
**Vital signs at admission** **Body temperature (^o^C)** **Respiratory rate (bpm)** **Hearth rate (bpm)** **MAP (mmHg)**	37 (36.8 – 37) 26 (22 – 30) 93 (80 – 103) 82 (73.5 – 94.8)	0.5195 0.0018 >0.9999 >0.9999	36.5 (36.3 – 37.2) 20 (16.7 – 21.7) 90 (75.7 – 99.7) 87 (80.7 – 88.7)	0.02 0.0518 0.7698 0.1820	37 (37 – 37.7) 24 (22 – 26) 84 (72 – 90) 75 (70.2 – 84.5)	0.2878 0.48 0.1295 0.3202

Data are displayed as n (%) or median (IQR). N is the total number of patients with available data. BMI, body mass index; bpm, breaths/beats per minute; COPD, chronic obstructive pulmonary disease; IQR, interquartile range; ICU, intensive care unit; MAP, mean arterial pressure; OSA, obstructive sleep apnea syndrome; SAH, systemic arterial hypertension; SD, standard deviation. Differences in continuous variables were estimated using the Kruskal Wallis with post hoc Dunn´s test. Differences in categorical variables were calculated using the Fisher’s exact or the Chi-square test as appropriate.

### Laboratory Parameters of Pandemic Influenza A(H1N1) and COVID-19

White blood cells (WBC), neutrophil counts, neutrophil to lymphocyte ratio (NLR), glucose, total bilirubin, and aspartate aminotransferase (AST) levels were similar in both pandemic influenza A(H1N1) and severe COVID-19 groups, but lower in the moderate COVID-19 group (**Table 2**). Low lymphocyte counts were observed among all participants, indicating that lymphopenia is not a unique feature of severe COVID-19. Renal function parameters did not differ between groups. However, levels of some tissue injury markers, such as alkaline phosphatase (ALP), alanine aminotransferase (ALT), lactate dehydrogenase (LDH), creatine phosphokinase (CPK), and procalcitonin, were higher in pandemic influenza A(H1N1) as compared to COVID-19 patients. We also observed that the SOFA and APACHE II scores were higher in pandemic influenza A(H1N1) patients. Importantly, both groups presented similar rates of complications, and received equal supportive medical interventions (**Table 3**). Despite this, the mortality of our cohort of critically ill pandemic influenza A(H1N1) patients was significantly lower (21%) than the mortality of severely ill COVID-19 patients (62%). No fatality cases were observed in the group of moderated COVID-19.

**Table 2:** Laboratory parameters of participants at admission.

Parameter	Influenza A(H1N1) pdm09A N = 23	*p*-value A vs. B	Moderate COVID-19B N = 10	*p*-value B vs. C	Severe COVID-19C N = 24	*p*-value A vs. C
**Glucose (mg/dL)**	132.1 (111 – 207.4)	0.1241	96.5 (85.2 – 112)	0.3025	124.3 (99 – 163.7)	>0.9999
**Blood count** **White blood cells (10^9^/L)** **Neutrophils (10^9^/L)** **Lymphocytes (10^9^/L)** **NLR** **Hgb (g/dL)** **Platelets (10^9^/L)**	7.3 (5.8 – 11.9) 5.7 (4.6 – 9.9) 0.7 (0.4 – 0.9) 8.4 (5.1 – 17.1) 14.2 (13 – 17.3) 173 (141 – 205)	0.0069 0.0057 0.5050 0.0120 0.6994 >0.9999	4.0 (3.5 – 5.7) 2.9 (1.8 – 3.9) 0.9 (0.6 – 1.2) 3.1 (1.6 – 5.7) 15.4 (14.2 - 16.4) 192 (137 – 224)	0.0007 0.0184 >0.9999 0.0702 0.0755 0.7794	9.5 (6.4 – 13.1) 7.4 (4.1 – 10.1) 0.9 (0.6 – 1.2) 8.7 (3.7 – 13.4) 13.7 (13.2 – 15.1) 208 (165 – 258)	>0.9999 >0.9999 0.3522 >0.9999 0.5663 0.1547
**Renal function** **Cr (mg/dL)** **BUN (mg/dL)** **Na (mmol/L)** **K (mmol/L)**	1.1 (0.9 – 2.2) 22.2 (16.2 – 34.3) 135.2 (132.5 – 139.3) 4.2 (3.8 – 4.6)	0.5773 0.1106 >0.9999 0.7951	1.0 (0.8 – 1.1) 14.5 (10.5 – 18.8) 137 (136 – 139) 4.0 (3.7 – 4.2)	>0.9999 0.6716 0.4082 0.6386	0.9 (0.8 – 1.5) 18 (11.9 – 26.8) 139.8 (136.2 – 141.7) 4.2 (4 – 4.5)	0.4344 0.7558 0.0161 >0.9999
**Liver function** **Total bilirubin (mg/dL)** **AST (U/L)** **ALT (U/L)** **ALP (U/L)**	0.6 (0.4 – 0.8) 60.9 (39.6 – 84.1) 29.2 (23.3 – 47.5) 122.7 (86.1 – 169.7)	0.0344 0.0026 0.4934 0.0016	0.3 (0.3 – 0.4) 22.6 (15 – 38.5) 21.7 (17.2 – 32.1) 72.5 (58.2 – 80.5)	0.0183 0.0170 0.0435 0.6821	0.5 (0.4 – 0.8) 43.5 (29 – 90.7) 40.2 (28.8 – 56.8) 78 (63.4 – 88.2)	>0.9999 >0.9999 0.5891 0.0104
**Other biomarkers** **LDH (U/L)** **CPK (U/L)** **Procalcitonin (ng/mL)**	643.8 (452.2 – 804.7) 274.4 (158.2 – 771.2) 0.6 (0.2 – 3.6)	<0.0001 0.0277 <0.0001	186 (165.8 – 251.5) 73 (49.7 – 161.3) 0.05 (0.05 – 0.08)	0.0070 0.1225 0.1652	414.5 (318 .4– 494.8) 160.3 (74.6 – 1419) 0.1 (0.09 – 0.17)	0.0269 >0.9999 0.0008
**Gasometric parameters** **pH** **PaO2 mmHg** **PCO2 mmHg** **Lactate (mmol/L)** **HCO3 (mEq/L)**	7.37 (7.32 – 7.45) 51 (38 – 67) 35 (30 – 48) 1.2 (0.8 – 1.5) 22.2 (17.9 – 27.4)	0.2325 0.0768 >0.9999 ND >0.9999	7.43 (7.41 – 7.46) 65 (55 – 91) 34 (29 – 37) ND 22.6 (21.5 – 25.8)	0.3277 0.1260 >0.9999 ND 0.4845	7.41 (7.33 – 7.45) 51 (42 – 65) 34 (27 – 47) 0.9 (0.8 – 1.1) 21.1 (19 – 22.8)	>0.9999 >0.9999 >0.9999 0.1309 0.9628
**PaO_2_/FiO_2_** **Mild (PaO_2_/FiO_2_ 201 - 300)** **Moderate (PaO_2_/FiO_2_ 101-200)** **Severe (PaO_2_/FiO_2_ <100)**	96 (62.8 – 160) 1 (4.34) 11 (47.82) 11 (47.82)	<0.0001 0.0002 0.3410 0.0129	314 (262 – 433) 7 (70) 3 (30) 0 (0)	0.001 0.0019 0.0836 0.0815	127 (96 – 155) 3 (12.5) 15 (62.5) 6 (25)	0.5999 0.6085 0.3118 0.1035
**Severity of illness scores** **SOFA** **APACHE II**	8 (7 – 13) 11 (5 – 18)	<0.0001 0.0405	1 (0 – 2) 4 (0 – 7.5)	0.0031 0.3546	6 (3 – 8) 7 (4 – 10)	0.0398 0.6420

Data are displayed as n (%) or median (IQR). N is the total number of patients with available data. ALP, alkaline phosphatase; APACHE-II, Acute Physiology and Chronic Health Evaluation II; AST, aspartate aminotransferase; ALT, alanine aminotransferase; BUN, blood ureic nitrogen; CPK, creatine phosphokinase; Cr, creatinine; FiO2, fraction of inspired oxygen; HCO3, bicarbonate; Hgb, hemoglobin; IQR, interquartile range; LDH, lactate dehydrogenase; ND, not determined; NLR, neutrophil/lymphocyte ration; PaO2, partial pressure of oxygen in arterial blood; PCO2, partial pressure of carbon dioxide in the blood; SD, standard deviation; SOFA, Sequential Organ Failure Assessment. Differences in continuous variables were estimated using the Kruskal Wallis with post hoc Dunn´s test. Differences in categorical variables were calculated using the Fisher’s exact or the Chi-square test as appropriate.

**Table 3:** Complications and treatment of study participants.

Parameter	Influenza A(H1N1) pdm09 A N = 23	*p*-value A vs. B	Moderate COVID-19 B N = 10	*p*-value B vs. C	Severe COVID-19 C N = 24	*p*-value A vs. C
**Complications** **Acute myocardial infarction** **Deep vein thrombosis** **Acute kidney injury** **Secondary infection**	3 (13.04) 1 (4.34) 10 (43.47) 14 (60.86)	0.5363 >0.9999 0.0148 0.0014	0 (0) 0 (0) 0 (0) 0 (0)	1 1 0.0815 0.0169	0 (0) 0 (0) 6 (25) 10 (41.66)	0.1092 0.4894 0.1814 0.1880
**Medical treatment** **Oseltamivir** **Antibiotic therapy** **No. of antibiotics per patient, median (range)** **Chloroquine/hydroxychloroquine** **Azithromycin** **Corticosteroids**	23 (100) 23 (100) 3.5 (2 – 10) 0 (0) 0 (0) 4 (17.39)	0.0012 1 0.0077 <0.0001 <0.0001 0.2890	6 (60) 10 (100) 2 (2 – 3) 10 (100) 10 (100) 0 (0)	0.9283 1 0.0252 <0.0001 0.0049 0.2908	14 (58.33) 24 (100) 4 (3 – 5) 23 (95.83) 13 (54.16) 5 (20.83)	0.0005 1 >0.9999 <0.0001 <0.0001 0.7643
**Respiratory support** **Nasal cannula** **MV** **Prone position** **ECMO**	0 (0) 23 (100) 14 (60.86) 2 (8.69)	<0.0001 <0.0001 0.0014 0.3360	10 (100) 0 (0) 0 (0) 0 (0)	<0.0001 <0.0001 0.0135 1	0 (0) 24 (100) 11 (45.83) 0 (0)	1 1 0.3017 0.2340
**Renal replacement therapy**	6 (26.08)	0.1445	0 (0)	0.2908	5 (20.83)	0.4252
**Mortality**	5 (21.73)	0.2911	0 (0)	0.0135	15 (62.5)	0.0077

Data are displayed as n (%) or median (IQR). N is the total number of patients with available data. ECMO, extracorporeal membrane oxygenation; IQR, interquartile range; MV, mechanical ventilation; SD, standard deviation. Differences in continuous variables were estimated using the Kruskal Wallis with post hoc Dunn´s test. Differences in categorical variables were calculated using the Fisher’s exact or the Chi-square test as appropriate.

### Immune Profiles of Pandemic Influenza A(H1N1) and COVID-19 Patients

**Figure 1:**
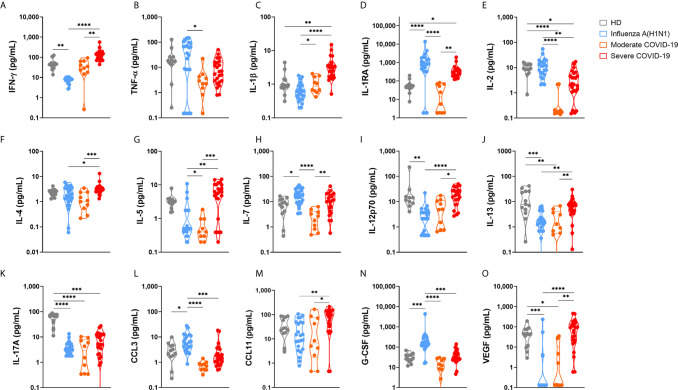
Serum cytokine levels in pandemic influenza A(H1N1) and COVID-19 patients. Serum levels of cytokines, chemokines, and growth factors in healthy volunteer donors (HD, n=13), patients with COVID-19 (n=10 moderate, 24 severe), and influenza (n=23), were assessed by Luminex assay. Violin plots display medians and interquartile ranges (IQR). Differences between groups we estimated using the Kruskal-Wallis test with *post hoc* Dunn´s test. Significant differences are denoted by bars and asterisks: *p ≤ 0.05, **p ≤ 0.01, ***p ≤ 0.001, ****p ≤ 0.0001. **(A)** IFN-γ, interferon-gamma; **(B)** TNF-α, tumor necrosis factor-alpha; **(C)** IL-1β, interleukin 1beta; **(D)** IL-1RA, interleukin 1 receptor antagonist; **(E)** IL-2, interleukin 2; **(F)** IL-4, interleukin 4; **(G)** IL-5, interleukin 5; **(H)** IL-7, interleukin 7; **(I)** IL-12p70, interleukin 12 p70 subunit; **(J)** IL-13, interleukin 13; **(K)** IL-17A, interleukin 17A; **(L)** CCL3, C-C motif chemokine ligand 3; **(M)** CCL11, C-C motif chemokine ligand 11; **(N)** G-CSF, granulocyte colony-stimulating factor; **(O)** VEGF, vascular endothelial growth factor.

The severity of pandemic influenza A(H1N1) and COVID-19 has been systematically attributed to an exacerbated production of pro-inflammatory cytokines (cytokine storm syndrome (CSS)) (25, 26). More recently, some researchers have also proposed that immune depression, rather than an exuberant immune activation, is responsible for the clinical pathology of severe COVID-19 (27). Comparing the immune responses elicited by SARS-CoV-2 and influenza A(H1N1) pdm09 virus may be more helpful in identifying unique immune mechanisms associated with morbidity and mortality in COVID-19. Thus, we determined the circulating levels of several immune mediators in pandemic influenza A(H1N1) and COVID-19 patients. Also, we correlated cytokine levels with clinical findings and disease outcomes. Our results showed that critically ill COVID-19 patients had increased serum levels of IL-1β, IL-1RA, IL-6, IL-9, and CXCL10, and lower levels of IL-2 and IL17A as compared to healthy volunteer donors (**Figure 1** and **Supplemental Figure 2**). These findings are coincident with the immune profiles that were reported in Chinese patients with COVID-19 (9, 17, 28). Levels of pro-inflammatory (IFN-γ, IL-1β, IL-6, IL-9, IL-12p70, CCL11) and anti-inflammatory (IL-4, IL-5, IL-10, IL-13) cytokines, as well as VEGF, were higher in severely ill COVID-19 patients as compared to pandemic influenza A(H1N1) subjects. In contrast, levels of IL-1RA, IL-2, TNF-α, CCL3, and G-CSF were more increased among pandemic influenza A(H1N1) patients (**Figure 1** and **Supplemental Figure 2**).

These serum cytokine profiles indicate that, besides a higher production of pro-inflammatory and Th1 cytokines, SARS-CoV-2, but not influenza A(H1N1) pdm09 infection, parallelly induces Th2 responses. This may suggest that a lack of sufficient regulation and balancing of the type of immune response triggered after SARS-CoV-2 infection might contribute to the immune dysfunction reported during COVID-19. Also, the proinflammatory and profibrotic immune profile observed in COVID-19 patients may contribute to the extensive tissue damage and poor outcomes reported during SARS-CoV-2 infection (27, 29). Other cytokines similarly increased in patients with severe pandemic influenza A(H1N1) and COVID-19 included IL-7, IL-15, IL8, and CXCL10 (**Supplemental Figure 2**).

### Histopathological Characteristics of the Lungs of Pandemic Influenza A(H1N1) and COVID-19 Patients

**Figure 2:**
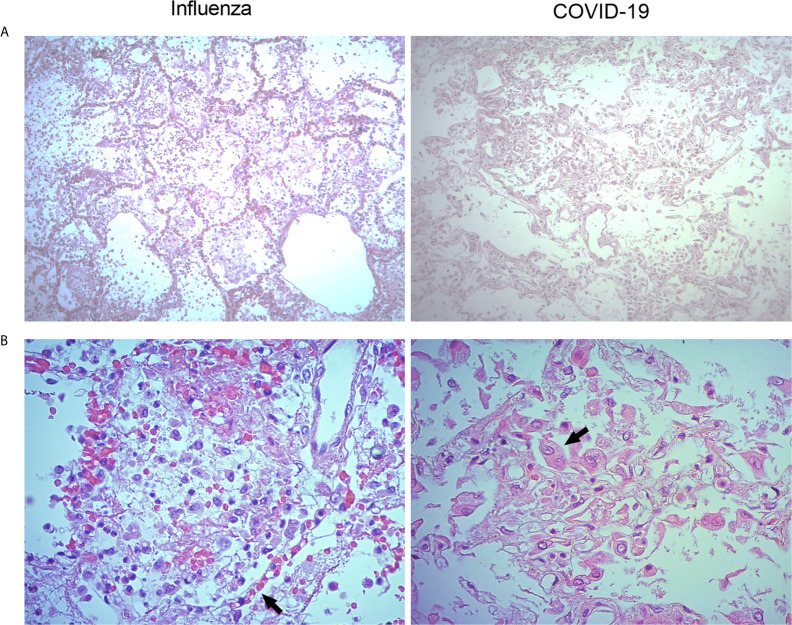
Histological characteristics of the lungs of patients with pandemic influenza A(H1N1) and COVID-19. Lung tissue autopsy specimens were obtained from patients that died of pandemic influenza A(H1N1) and COVID-19. **(A)** The histological changes induced in the lungs during pandemic influenza A(H1N1) were mainly characterized by intra-alveolar inflammatory infiltrates that did not compromise the integrity of alveolar walls (left panel). Meanwhile, the morphological changes of COVID-19 consisted of extensive inflammation, thickening of the alveolar walls, and partial loss of the histological architecture (right panel). H&E staining, x100. **(B)** The inflammatory infiltrates observed in the lungs of pandemic influenza A(H1N1) patients consisted of macrophages, polymorphonuclear cells, and scarce lymphocytes scattered between areas of edema, hemorrhage, and fibrin deposits. Also, congestive, and vasodilated capillaries (arrow) were observed in the alveolar walls of influenza patients (left panel). Conversely, the inflammatory infiltrates found in the lung of COVID-19 patients were dominated by macrophages. Furthermore, the detachment of alveolar epithelial cells, which showed atypical characteristics such as large nucleoli (arrow), was also notable in COVID-19 patients (right panel). H&E staining, x400.

Parallel histopathological comparative analyses of the lungs of COVID-19 and pandemic influenza A(H1N1) patients have not been conducted. Here, we obtained lung autopsy specimens from individuals that succumbed to either of these diseases and analyze their pathological features. Our analysis revealed that pandemic influenza A(H1N1) induces alveolar edema and intra-alveolar inflammatory infiltrates in the lungs, sparing the integrity of alveolar walls and the micro-architecture of the organ (**Figure 2A**, left panel). These findings are compatible with a typical pattern of alveolar pneumonia. The inflammatory infiltrates observed in the lungs of pandemic influenza A(H1N1) patients were composed of macrophages, polymorphonuclear cells, and scarce lymphocytes scattered between areas of intra-alveolar edema, hemorrhage, and fibrin mucoid exudates. Furthermore, although conserved, the alveolar walls showed capillaries with vasodilation and congestion (**Figure 2A**, right panel). Meanwhile, SARS-CoV-2 induced intense and extensive inflammatory lung infiltrates, as well as thickness of alveolar walls, hemorrhages, and partial loss of the histological architecture of the lung. These changes are compatible with interstitial pneumonia (**Figure 2B**, left panel). The inflammatory infiltrates observed in the lungs of COVID-19 patients were mainly composed of macrophages. Notably, the lungs infected with SARS-CoV-2 showed scarce lymphocytes and detachment of pneumocytes, which showed hyperplasia, cellular changes, and prominent nucleoli (**Figure 2B**, right panel).

**Figure 3:**
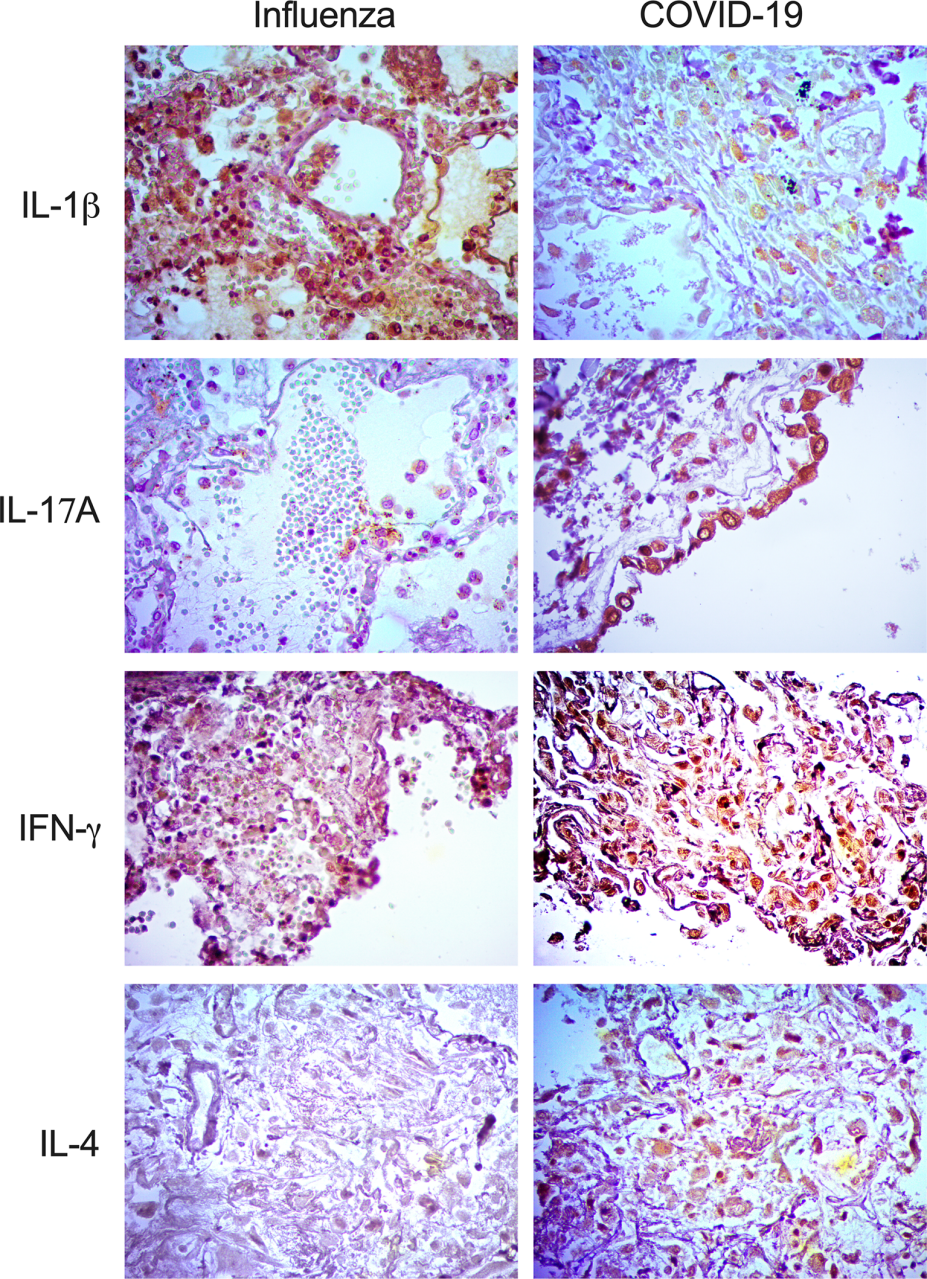
Expression of immune markers in the lungs of pandemic influenza A(H1N1) and COVID-19 patients. Expression of different immune markers in lung autopsy specimens from pandemic influenza A(H1N1) and COVID-19 patients was assessed using specific antibodies by immunohistochemistry (IHQ), x400.

Interestingly, our IHQ analysis showed that IFN-γ, IL-1β, and IL-17A were expressed in the lungs of patients with both diseases, mainly inside macrophages and pneumocytes (**Figure 3**). However, the intensity of expression of IFN-γ and IL-17A was higher in patients infected with SARS-CoV-2. Strikingly, IL-4, a Th2 cytokine, was absent in the lungs of pandemic influenza A(H1N1) patients but expressed in COVID-19 subjects (**Figure 3**). These findings are in line with the combined Th1/Th2 immune profile detected only in the serum of our cohort of patients infected with SARS-CoV-2 but not in pandemic influenza A(H1N1) subjects.

### Clinical and Immunological Markers Distinguishing Pandemic Influenza A(H1N1) and COVID-19

**Figure 4:**
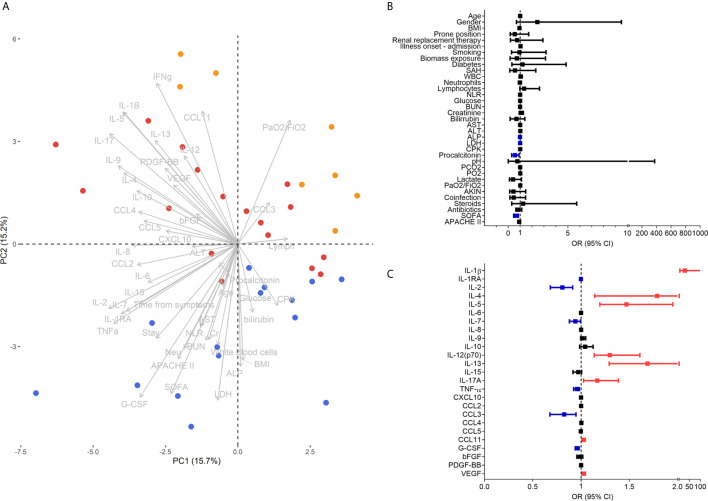
Clinical and immunological factors that distinguish pandemic influenza A(H1N1) and severe COVID-19. **(A)** Principal component analysis (PCA) of the clinical and immunological characteristics of study participants. Each dot represents a single individual, and each color represents a group of participants: blue for pandemic influenza A(H1N1), orange for moderate COVID-19, and red for severe COVID-19. **(B, C)** Bivariate logistic regression analysis of the clinical and immunological characteristics associated with the causative pathogen in the two cohorts of patients with severe influenza and COVID-19. The forest plots show the odds ratio (OR) and 95% CI interval values that were non-significant (black) and significant for severe COVID-19 (red). OR values of factors inversely associated with severe COVID-19 that instead predict pandemic influenza A(H1N1) are shown in blue color. Absolute OR values are also presented in **Supplementary Table 3**.

To determine which clinical and immunological characteristics contributed more to the differences between pandemic influenza A(H1N1) and COVID-19, we performed PCA. The analysis showed that pandemic influenza A(H1N1) patients cluster apart from the combined cohort of COVID-19 subjects in the PC2 (**Figure 4A**). Of note, clinical characteristics contributed to 31.2% of the total variance explained by the two first PCs (12.51% to PC1 and 50.03% to PC2). Meanwhile, serum cytokine levels contributed to 68.7% of the total variance explained by the two first PCs (87.48% to PC1 and 49.96% to PC2). These data indicate that immunological characteristics may be more useful than clinical variables to discriminate between both diseases. Thus, we performed additional PCAs using only clinical or immunological characteristics. We observed that patients with severe pandemic influenza A(H1N1) were not separated from severely ill COVID-19 patients by their clinical features, but they clustered apart from moderate COVID-19 subjects (**Supplemental Figure 3a**). Age, neutrophils, ALP, CPK, bilirubin, LDH, PaO2/FiO2, and SOFA were the clinical variables that contribute more to the first two PCs of this analysis. Conversely, pandemic influenza A(H1N1) patients clustered apart from the entire COVID-19 cohort in a PCA using only serum cytokine levels (**Supplemental Figure 3b**). IFN-γ, IL-1RA, IL-5, IL-9, IL-10, and G-CSF levels contribute to the first two PCs of this PCA.

Using logistic regression analyses, we further evaluated which clinical and immune factors differentiate our two cohorts of severely ill pandemic influenza A(H1N1) and COVID-19 patients. IFN-γ was not included in this analysis, as it showed perfect discrimination of severe COVID-19 from pandemic influenza A(H1N1). We identified that LDH, ALP, procalcitonin, SOFA score, IL-1RA, IL-2, IL-7, TNF-α, CCL3, and G-CSF levels were significantly associated with severe pandemic influenza A(H1N1). In contrast, IL-1β, IL-4, IL-5, IL-12p70, IL-13, IL-17A, CCL11, and VEGF levels predicted severe COVID-19 (**Figures 4B, C**). Some of these factors, along with PaO2/FiO2 index, the incidence of acute kidney injury (AKIN), co-infections, APACHE-II score, IFN-γ, IL-15, and CCL5, also contributed to differentiate the entire COVID-19 cohort from pandemic influenza A(H1N1) subjects (**Supplemental Figure 4**).

**Figure 5:**
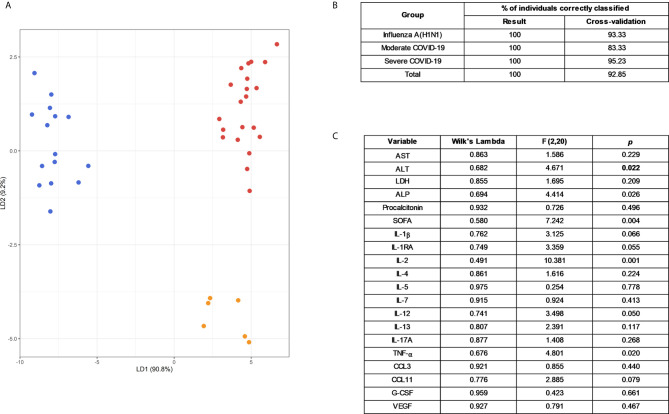
Selected clinical and immunological characteristics that better distinguish pandemic influenza A(H1N1) from COVID-19. **(A)** Linear discriminant analysis (LDA) plot of the first two discriminant functions showing the separation of the different groups of study participants according to a set of selected clinical and immunological characteristics used in combination. Each dot represents a single individual, and each color represents a group of participants: blue for pandemic influenza A(H1N1), orange for moderate COVID-19, and red for severe COVID-19. **(B)** Accuracy of the LDA results before and after a “leave-one-out” cross-validation. **(C)** The discriminant potential of each individual variable included in the LDA was estimated using the Wilk´s Lambda test. The table displays values of Wilk´s lambda, F, and *p* for each variable.

**Figure 6:**
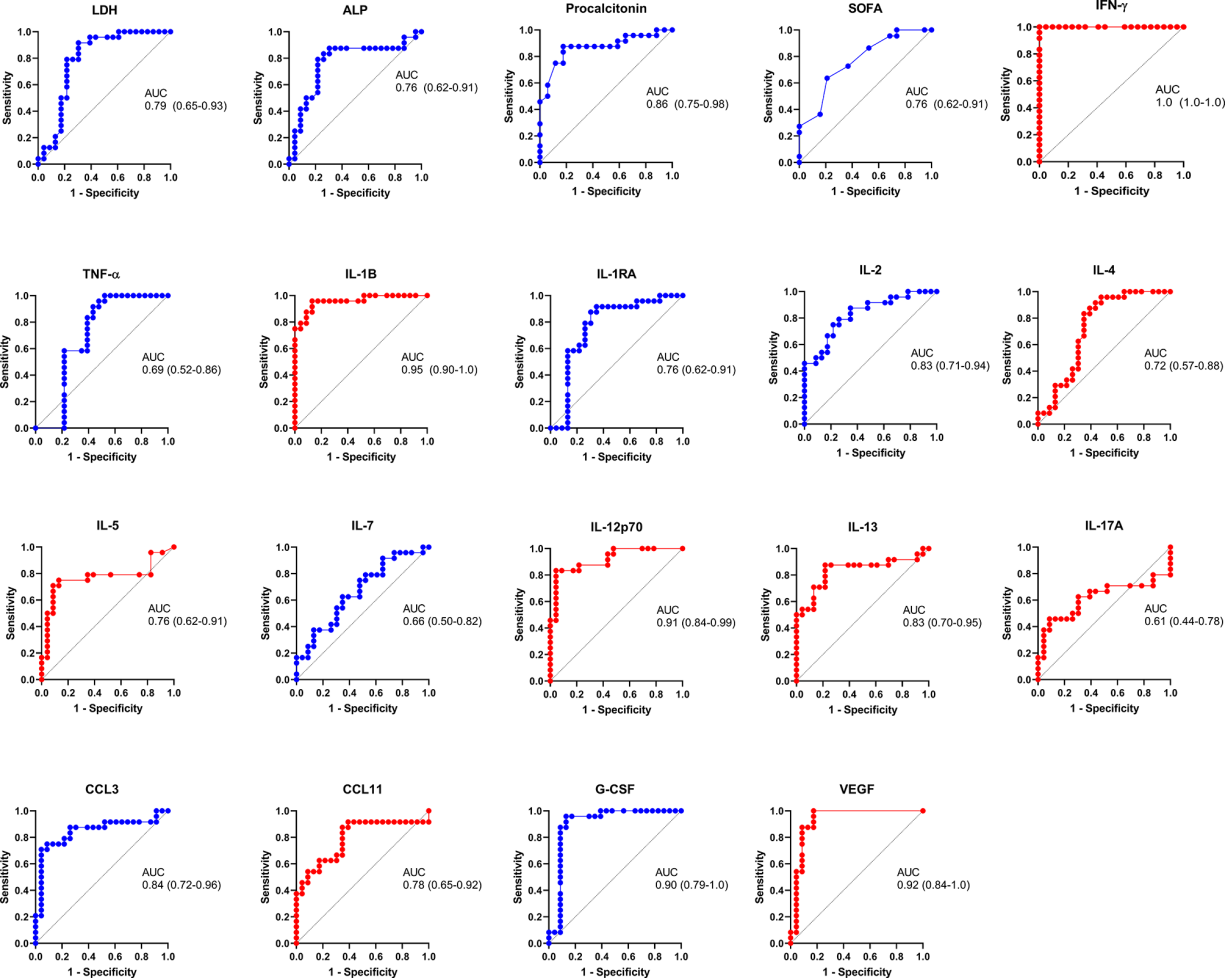
Diagnostic value of the clinical and immunological factors that distinguish between severe COVID-19 and pandemic influenza A(H1N1). Receiver operating characteristic (ROC) curves were constructed for the clinical and immunological characteristics that showed significant OR values in the bivariate logistic regression analysis. ROC curves of variables associated with pandemic influenza A(H1N1) are shown in blue color, whereas variables associated with severe COVID-19 are displayed in red color. The graphs show area under the curve (AUC) and 95% CI interval values.

An LDA showed that some of these selected parameters, along with AST and ALT, used together, accurately differentiate between severe pandemic influenza A(H1N1), moderate COVID-19, and severe COVID-19 groups (**Figures 5A, B**). Since it would be impractical to assess all these factors combined to differentiate both diseases, we analyze the results of the LDA using the Wilk´s Lambda test. This analysis showed that ALT, ALP, SOFA, IL-2, and TNF-α were crucial for the discriminative power of our LDA model (**Figure 5C**). Furthermore, receiver operating characteristics (ROC) curve analyses showed that IFN-γ, IL-1β, IL-12p70, G-CSF, and VEGF had the highest diagnostic performance to distinguish severe COVID-19 and pandemic influenza A(H1N1) (**Figure 6**).

### Clinical and Immunological Prognostic Factors in Pandemic Influenza A(H1N1) and COVID-19

**Figure 7:**
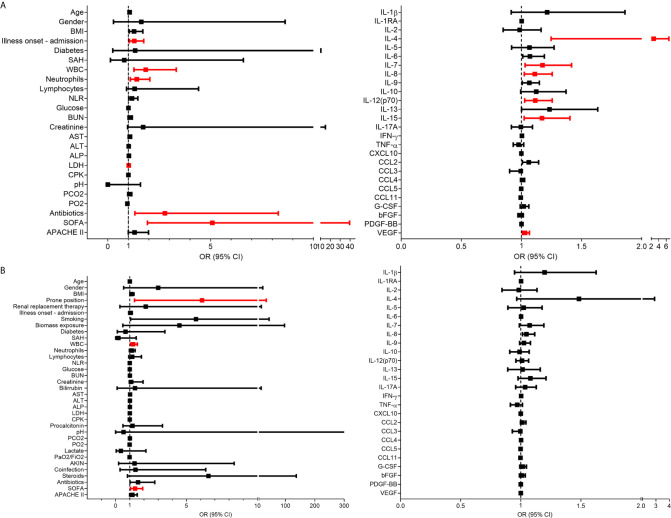
Clinical and immunological factors associated with disease outcomes in patients with COVID-19. **(A)** Bivariate logistic regression analysis of the clinical and immunological characteristics associated with intubation in patients COVID-19. **(B)** Clinical and immunological factors associated with mortality in patients with COVID-19. The forest plots show the odds ratio (OR) and 95% CI interval values. OR values that did not include the null value in the 95% CI were considered significant for intubation/mortality and are shown in red color. Absolute OR values are also presented in **Supplemental Table 5**.

We also evaluated the prognostic value of clinical and immunological factors in pandemic influenza A(H1N1) and COVID-19. Among COVID-19 patients, the duration of symptoms before admission, WBC, neutrophil counts, LDH, and SOFA score predicted severe disease defined as the need for intubation (**Figure 7A**). IL-4, IL-7, IL-8, IL-12p70, IL-15, and VEGF were also associated with increased risk of intubation in COVID-19 subjects. IL-6 showed increased but not significant OR values for severity in the combined COVID-19 cohort, contrasting with previous studies that indicate that IL-6 is significantly associated with severe COVID-19 (18, 30). Using a similar approach, we observed that WBC, and SOFA score conferred a higher risk of death after SARS-CoV-2 infection in the entire cohort of COVID-19 patients (**Figure 7B**). Likewise, the need for renal replacement therapy (OR 32, 3 – 849.9 95% CI, *p* = 0.0029), and the use of steroids (OR 25.5, 2.1 – 698.4 95% CI, *p* = 0.0091), were associated with mortality risk after pandemic influenza A(H1N1) (**Supplemental Figure 5**), as reported before (31, 32). However, none of the evaluated cytokines were associated with mortality in COVID-19 and pandemic influenza A(H1N1) patients (**Figure 7** and **Supplemental Figure 5**). At the time of patient recruitment, there was no consensus regarding the use of steroids for COVID-19, and the RECOVERY trial had not been published (33). Hence, only some of our COVID-19 patients were treated with steroids.

### Additional Immune Markers Distinguishing Pandemic Influenza A(H1N1) From COVID-19

**Figure 8:**
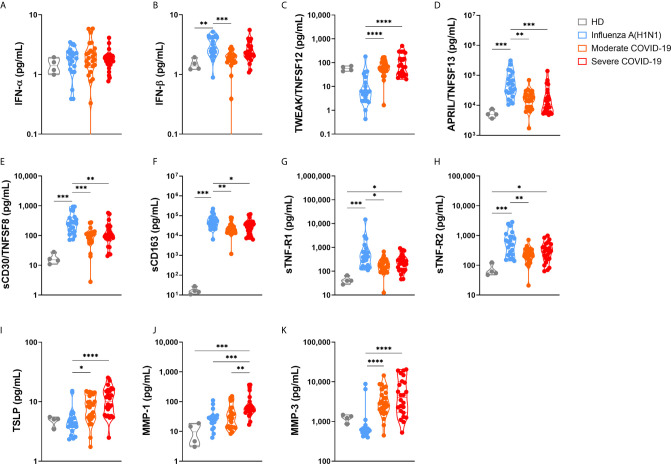
Immune mediators in the plasma of patients with pandemic influenza A(H1N1) and COVID-19. Levels of different soluble immune mediators in plasma samples from patients with COVID-19 (n=25 moderate, 24 severe) and pandemic influenza A(H1N1) (n=23), as well as in samples from healthy volunteer donors (HD, n=4) were assessed by Luminex assay. Violin plots display medians and interquartile ranges (IQR). Differences between groups we estimated using the Kruskal-Wallis test with *post hoc* Dunn´s test. Significant differences are denoted by bars and asterisks: *p ≤ 0.05, **p ≤ 0.01, ***p ≤ 0.001, ****p ≤ 0.0001. **(A)** IFN-α, interferon-alpha; **(B)** IFN-β, interferon-beta; **(C)** TWEAK/TNFSF12, tumor necrosis factor-like weak inducer of apoptosis/tumor necrosis factor ligand superfamily member 12; **(D)** APRIL/TNFSF13, A proliferation-inducing ligand/tumor necrosis factor ligand superfamily member 13; **(E)** sCD30/TNFRSF8, soluble CD30/tumor necrosis factor ligand superfamily member 8; **(F)** sCD163, soluble CD163; **(G)** sTNF-R1, soluble tumor necrosis factor receptor 1; **(H)** sTNF-R2, soluble tumor necrosis factor receptor 2; **(I)** TSLP, thymic stromal lymphopoietin; **(J)** MMP-1, metalloprotease 1; **(K)** MMP-3, metalloprotease 3.

Finally, we analyzed another set of immune mediators in the blood of 25 moderate and 24 severe COVID-19 patients, as well as in 22 pandemic influenza A(H1N1) subjects, from which we were able to obtain plasma samples (**Figure 8** and **Supplemental Figure 6**). Plasma levels of these factors showed only a few correlations with clinical characteristics and serum cytokine levels (**Supplemental Figure 7**). The overall profile of these correlations was different in pandemic influenza A(H1N1) and COVID-19 patients, suggesting distinct immune mechanisms underlying clinical manifestations of both diseases.

Although levels of plasma type I interferons were below the levels of reliable detection, IFN-α, and IFN-β were increased among all participant groups as compared to healthy controls (**Figure 8**). Furthermore, a slight increase in the levels of IFN-β was noticed in pandemic influenza A(H1N1) patients as compared to COVID-19 patients. Remarkably, although elevated, the levels of APRIL/TNFSF13, sCD30, sCD163, sTNF-R1, and sTNF-R2 were lower in COVID-19 than in pandemic influenza A(H1N1) patients. APRIL/TNFSF13 is crucial for plasma cell survival (34). Thus, plasma cell responses could be downregulated in COVID-19 as compared to pandemic influenza A(H1N1). Soluble CD30 has been proposed as a marker of T cell activation during solid organ transplant rejection (35), whereas sCD163 is a readout of macrophage activation (36). Hence, our data may indicate a depletion of activated lymphocytes and macrophages from the circulation during SARS-CoV-2 infection, despite the high levels of inflammatory mediators found in COVID-19 patients. Soluble TNF-R1 and sTNF-R2 act as decoy receptors for TNF-α (37); as such, patients with COVID-19 might be less capable of balancing pathogenic TNF-α activities than individuals with pandemic influenza A(H1N1).

TWEAK, TSLP, MMP-1, and MMP-3 were elevated in COVID-19 cases. TWEAK is a stimulator of IL-6, IL-8, CXCL10, and MMP-1 (38, 39). As such, high levels of TWEAK might expand the inflammatory response observed in COVID-19 patients. TSLP is a promoter of allergic inflammation and Th2 responses (40). Indeed, high TSLP levels coincide with a Th2 cytokine profile in our COVID-19 cohort. Our results also indicate a possible role for MMP-1 and MMP-3 in lung injury associated with COVID-19, two matrix metalloproteases implicated in tissue damage underlying other lung diseases (41–43).

## Discussion

The ongoing winter in the Northern hemisphere has been one of the most challenging public health crises in recent history due to the convergence of influenza and COVID-19. This situation could be further aggravated at settings of high pandemic influenza A(H1N1) circulation. Thus, a better understanding of the clinical and immunopathological characteristics that differentiate both diseases is still required to guide specific therapeutic approaches. This includes the selection of adequate antiviral drugs and appropriate immunological therapeutics for each case. Unfortunately, whereas our knowledge of the immunopathogenesis of pandemic influenza A(H1N1) has improved over the last decade, the current lack of understanding of the COVID-19 pathobiology remains incomplete. This is a barrier to the identification of targets for drug and vaccine development. The inevitable co-circulation of influenza viruses and SARS-CoV-2 and the potential scenarios of viral co-infection may further represent an aggravation of the COVID-19 morbidity and mortality. However, we do not know if an infection with SARS-CoV-2 in patients already infected with influenza viruses would result in worse or better clinical outcomes. The outcomes of the opposite scenario are also speculative. Despite this, it is essential to have reliable indicators to differentiate these conditions, especially in settings of limited resources to perform RT-PCR tests.

Some recent literature reviews have tried to highlight differences between patients infected with SARS-CoV-2 and seasonal influenza viruses (14, 15). However, these retrospective comparisons carry the risk of biased conclusions due to differences in the genetic background, sociocultural characteristics, and access to medical attention of populations from different regions. Thus, parallel comparisons of influenza and COVID-19 cases in geographical settings with similar health care resources would provide a better perspective of the main differences between these entities. In this context, Mexico is an ideal place to conduct comparative studies between pandemic influenza A(H1N1) and COVID-19, as this country was the site of origin of the influenza A(H1N1) pdm09 virus (2–4). Since its emergence in 2009, hospitals around Mexico have acquired ample experience in the management of severe cases of this viral infection, which has resulted in progressive decreases in mortality rates over the last ten years (44). On February 28^th^, 2020, Mexico confirmed its first two cases of SARS-CoV-2. Ever since, the epidemiological curve of COVID-19 shows a continuous increase in the number of positive cases, with more than 2.2 million cases and 207,000 deaths reported on March 2^nd^ of 2021 (45).

Here, we compared the clinical, histopathological, and immune characteristics of pandemic influenza A(H1N1) and COVID-19 patients. One of the most striking findings of our study was that most of the clinical and laboratory parameters routinely evaluated in emergency departments were similar between both infections in severe disease. Nonetheless, some features separated well moderate COVID-19 patients from severe COVID-19 and pandemic influenza A(H1N1) subjects. Interestingly, our data reveal that respiratory symptoms are more common during pandemic influenza A(H1N1), whereas dry cough and gastrointestinal symptoms are distinctive characteristics associated with COVID-19. These clinical differences may traduce distinct infective capacities of both viruses to affect several organs besides the lungs. In this sense, influenza viruses are thought to be primary respiratory pathogens that rarely cause extrapulmonary dissemination (46). Meanwhile, it is accepted that SARS-CoV-2 has a broad infective capacity to invade several tissues and organs (47). The expression of the angiotensin I converting enzyme 2 (ACE2), the transmembrane serine protease 2 (TMPRSS2), furin, cathepsin L, and other viral entry factors in human organs determine the tissue tropism of SARS-CoV-2. These factors are expressed in the lungs; nonetheless, their expression is even higher at several parts of the upper and lower gastrointestinal tract (48). This might explain the clinical differences observed in our study.

We also found that levels of ALP, ALT, LDH, CPK, procalcitonin, as well as SOFA and APACHE II scores were higher in pandemic influenza A(H1N1) as compared to both groups of COVID-19 patients. Meanwhile, the PaO2/FiO2 upon arrival was similar in severe COVID-19 and severe pandemic influenza A(H1N1) patients. These findings coincide with the results of a previous study evaluating the differences in clinical presentations between Chinese ARDS patients infected with either SARS-CoV-2 or influenza A(H1N1) (13). The researchers also found that ground-glass opacities were more common in radiological studies of COVID-19 patients, whereas consolidation opacities were more frequent in influenza subjects. Ground-glass opacities are typically associated with an interstitial inflammatory process of the lung, whereas consolidations traduce intra-alveolar exudates (49). Here, we found that the histopathological pattern induced after lung infection with SARS-CoV-2 is mainly characterized by an interstitial inflammatory infiltrate. Meanwhile, pandemic influenza A(H1N1) induces changes compatible with alveolar pneumonia. Together, both studies highlight that the two diseases display crucial differences in the histological characteristics of the infected lungs that may also translate into distinctive clinical manifestations.

The immune response against SARS-CoV-2 is not well comprehended so far. The prevailing paradigm to explain the morbidity and mortality of COVID-19 patients is that SARS-CoV-2 elicits an exuberant immune reaction characterized by a dysregulated cytokine production. This phenomenon, known as “cytokine storm,” is thought to be responsible for mediating tissue injury in patients with COVID-19 that progress to severe illness (19, 28, 50, 51). The immune receptors that recognize the viral infection and initiate the immune responses against SARS-CoV-2 are unknown. As this virus is genetically related to SARS-CoV-1, it is presumed that both viruses share mechanisms of infection. In this sense, SARS-CoV-1 is recognized by the toll-like receptors (TLR) TLR3 and TLR4, which induce an immune reaction *via* MyD88 and TRIF pathways (52, 53). Furthermore, SARS-CoV-1 triggers the production of IL-1β through the activation of the inflammasome (54). It is also possible that SARS-CoV-2 activates the inflammasome, as high levels of IL-1β have been observed in COVID-19 patients (55). Other immune mediators exaggeratedly produced in response to SARS-CoV-2 include IL2, IL-6, IL7, IL10, G-SCF, CXCL10, CCL2, CCL3, and TNF-α (9, 17, 28). Similar immune signatures were detected in our cohort of COVID-19 patients. Strikingly, our study, and two recent investigations carrying out single cell RNA sequencing of immune cells and cytokine determinations in BAL (16, 56), converge in a major pathogenic role of IL-1 β, IL-6, and CCL2 in patients who develop severe COVID-19 compared to people with less severe disease.

Meanwhile, the pathogenicity and virulence of the influenza A(H1N1) pdm09 virus are due to acquired properties contributing to alter the regulation of inflammatory responses and evade antiviral immunity. Previously, we have described that pandemic, but not seasonal influenza A strains, downregulate the expression of the suppressors of cytokine signaling 1 (SOCS-1) and increase the production of IL-6, IL-8, TNF-α, IL-10, CCL3, CCL4, and CCL5 in experimental infection assays of human lung A549 epithelial cells and human macrophages (57). Levels of IL-6, IL-8, TNF-α, and CCL3 were also increased in our cohort of pandemic influenza A(H1N1) patients, validating our previous observations. The influenza A(H1N1) pdm09 also suppresses the expression of the retinoid-inducible gene I (RIG-I) and induces lower levels of type I interferons in human macrophages and human lung epithelial cells, as compared to seasonal influenza A strains (57). In this sense, it is possible that blocking type I interferon responses might be a strategy of SARS-CoV-2 to evade antiviral immune mechanisms, as we found very low induction of plasma IFN-α and IFN-β in both pandemic influenza A(H1N1) and COVID-19 patients. A similar type I interferon deficiency was observed in the blood of French critically ill COVID-19 patients (58). Conversely, another study from Korea reveals that type I interferon expression is increased in BAL immune cells from severe COVID-19 patients (16), indicating that antiviral interferon responses against SARS-CoV-2 might be highly compartmentalized into the lungs and barely detectable in the blood.

Notably, despite the dysregulated production of other immune mediators, an ample range of immune cell subtypes are depleted from the circulation of patients with severe SARS-CoV-2 infection. These cells include monocytes, dendritic cells, CD4+ T cells, CD8+ T cells, B cells, and NK cells (59). Furthermore, the few adaptive lymphocytes that remain in the blood express markers of functional exhaustion (29). These data suggest that severe COVID-19 is a state of immunosuppression similar to the known sepsis‐induced immunosuppression (60). Notably, a recent study by Remy and collaborators has shown that the immunosuppression observed in COVID-19 is even more profound than in critically ill patients with sepsis of other causes (27). These researchers demonstrated that the production of IFN-γ by peripheral blood T cells of COVID-19 patients was impaired as compared with T cells from healthy individuals and septic patients after anti-CD3/anti-CD28 antibody stimulation. Furthermore, a reduced production of TNF-α by stimulated monocytes from COVID-19 patients was noticed. These findings led the researchers to propose that the primary immune mechanism underlying the morbidity and mortality of COVID-19 is immunosuppression rather than hyperinflammation.

In this context, our study confirms that the immune response against SARS-CoV-2 is entirely different from the response against pandemic influenza A(H1N1). Indeed, our analyses bring forward a set of immunological markers with the potential to differentiate COVID-19 from pandemic influenza A(H1N1) successfully. Measuring some of these markers might improve the diagnostic approach and subsequent therapeutic decision for ARDS patients. Also, our study may provide additional evidence useful to clarify current controversies about the immunopathology of COVID-19. Based on our results and previous investigations, we propose that hyperinflammation and immunosuppression are not mutually exclusive in COVID-19. First, our data showed some indirect readouts of immunosuppression in individuals infected with SARS-CoV-2. For instance, we found that TNF-α levels were lower in the serum of COVID-19 patients as compared to pandemic influenza A(H1N1) patients. This coincides with the limited capacity of monocytes from COVID-19 patients to produce TNF-α upon stimulation described by Remy et al. (27). We also observed lower plasma levels of the macrophage activation marker sCD163, although macrophages infiltrating the lungs of COVID-19 patients expressed several cytokines. Furthermore, we found low levels of IL-2 and APRIL/TNFSF13 (two immune mediators crucial for T-cell and plasma cell survival), as well as sCD30 (a marker of lymphocyte activation) in the circulation of COVID-19 but not pandemic influenza A(H1N1) patients. Similarly, we observed a lack of lymphocytes in the inflammatory infiltrates found in lung autopsy specimens from patients that died of COVID-19. These findings may reflect a depletion of activated lymphocytes and monocytes from the circulation during SARS-CoV-2 infection and poor recruitment of lymphocytes to the lungs.

At the same time, we have described that an exacerbated polyfunctional immune response prevails in the circulation of COVID-19 patients. Such a response is characterized by higher levels of Th1 as well as Th2 cytokines as compared to pandemic influenza A(H1N1) patients. Conversely, although pandemic influenza A(H1N1) subjects also display elevated levels of some inflammatory mediators, these individuals may have enough regulatory mechanisms that counteract the detrimental effects of hyperinflammation. The higher levels of IL-1RA observed here in pandemic influenza A(H1N1) patients as compared to COVID-19 subjects well exemplify this. Furthermore, we found higher serum levels of the C-X-C motif chemokine ligand 17 (CXCL17), a mucosal chemokine with anti-inflammatory properties, in pandemic influenza A(H1N1) but not COVID-19 patients (61). In addition, the serum cytokine pattern of COVID-19 resembles the inflammatory profile of rheumatoid arthritis patients with interstitial lung disease (62), and the polyfunctional inflammatory response of the cytokine release syndrome (CRS) that occurs after chimeric antigen receptor (CAR) T-cell therapy (63). Immunosuppression and hyperinflammation are also a hallmark of both of these conditions.

Of note, the higher levels of Th2 cytokines, particularly IL-4 and IL-5, might inhibit Th1 protective antiviral responses in COVID-19 patients. Thus, our data indicate that a lack of immune balance of the type of effector response is another crucial determinant of the collapse of the host protective immunity against SARS-CoV-2. This Th2 biased response may generate interstitial infiltrates of Th2 cells, neutrophils, eosinophils, and type 2 innate lymphoid cells, mediating lung inflammation, and tissue damage. In fact, critically ill COVID-19 patients usually show interstitial lung infiltrates, some of which resemble several forms of progressive interstitial lung disease like cryptogenic organizing pneumonia and non-specific interstitial pneumonia (9, 64–66). Here, we also observed interstitial inflammation and expression of IL-4 in the lungs of COVID-19 patients but not pandemic influenza A(H1N1) subjects. These deleterious effects of Th2 responses could also explain the abnormalities in lung function, and progression to pulmonary fibrosis observed in more than 45% of COVID-19 patients discharged from hospitals (67), particularly in older patients. Hence, it would be of great interest to characterize the cytokine profile of COVID-19 patients that subsequently develop any form of interstitial lung disease, as they would benefit from specific and anti-fibrotic therapeutics.

We propose that ideal immune therapeutics for COVID-19 should be directed not only to blocking or enhancing specific immune signaling pathways to counteract hyperinflammation or reverting immunosuppression. Instead, immune therapies must re-establish a convenient immune balance that promotes protective immunity against SARS-CoV-2. Under the light of this hypothesis, several immune mediators and immune cell subsets could be targeted. For instance, type 2 innate lymphoid cells (ILC2s) have been identified as the leading producers of Th2 cytokines in the lungs, contributing to potent allergen-induced airway inflammation even in lymphopenic hosts (68). Thus, ILC2s may constitute novel targets to inhibit Th2 responses in COVID-19 patients. The potential pathogenic effects of Th2-biased responses in COVID-19 may also be counteracted with monoclonal antibodies. For instance, dupilumab, a monoclonal antibody against IL-4, has been safely used in patients with atopic dermatitis and COVID-19, without increased risk of severe complications of the infection. Remarkably, some patients receiving dupilumab that later acquired the infection with SARS-CoV-2 did not show respiratory symptoms (69–71). Finally, TSLP could be another target to inhibit Th2 responses in COVID-19 patients, as this molecule promotes allergic inflammation (40), and indeed, high levels of TSLP were observed in our cohort of COVID-19 but not pandemic influenza A(H1N1) subjects.

## Limitations

A limitation of our study is that we did not recruit patients infected with seasonal influenza virus subtypes. Thus, our observations are only useful to distinguish between influenza A(H1N1) pdm09 and SARS-CoV-2 infection. The clinical and immunological characteristics of SARS-CoV-2 and seasonal influenza have been compared in a recent study by Mudd et al. (72). In such a study, researchers found that COVID-19, as compared to seasonal influenza, is characterized by lower mean cytokine levels in serum. Conversely, we found that cytokine levels were higher in COVID-19 patients than in individuals with pandemic influenza A(H1N1). These discrepancies are probably related to variations in the virulence and capacity to induce inflammatory immune responses of seasonal and pandemic influenza viruses. Lee et al. (16), also compared single cell RNA sequencing of BAL immune cells from COVID-19 and influenza A patients. Although these researchers did not specify the subtype of influenza A virus infection, their results coincide with our data with regards to the higher induction of IL-1β in COVID-19 than influenza. However, differential roles of TNF and type I interferon signaling during the two diseases are important discrepancies between their and our study. The source and time of sample collection can potentially be a reason for these differences. Finally, another limitation of our study is that we did not measure cytokine levels in serial serum/plasma samples from our two cohorts of pandemic influenza A(H1N1) and COVID-19 patients. Thus, future investigations should compare differences in the kinetics of immune responses against both diseases. Despite this, our study provides important insights into the differences between the two most important respiratory pathogens that have caused pandemics of international concern in recent years.

## Conclusions

In conclusion, our results demonstrate significant differences in the immune responses elicited after SARS-CoV-2 and influenza A(H1N1) pdm09 virus. Our data support the use of specific clinical characteristics, laboratory parameters, and immunological markers to differentiate SARS-CoV-2 infection from pandemic influenza A(H1N1). These data may also contribute to the discovery of novel therapeutic targets to counteract harmful immune mechanisms underlying the immunopathology of COVID-19 and pandemic influenza A(H1N1).

## Data Availability Statement

The raw data supporting the conclusions of this article will be made available by the authors, without undue reservation.

## Ethics Statement

The studies involving human participants were reviewed and approved by Institutional Review Boards of the Instituto Nacional de Ciencias Médicas y Nutrición Salvador Zubirán (INCMNSZ, approval number: 3349) and the Instituto Nacional de Enfermedades Respiratorias Ismael Cosío Villegas (INER, approval number: B28-16 and B09-20). The patients/participants provided their written informed consent to participate in this study.

## Author Contributions 

Design of the research study: JC-P, TR-R, SK, AZ, and JZ. Recruited patients: JC-P, TR-R, MS-V, DH-G, EM-G, ES, JM-R, JB-R, HV-R, G-CS, NA-P, DG-C, GH, JG, LM-H, LP-B, GD-C, and CH-C. Retrieved clinical data: JC-P, TR-R, MS-V, DH-G, N-AP, GH, LM-H, CH-C, AH-M, and LO. Collected and processed blood samples: JC-P, LJ-A, AC-L, TR-R, GR-M, EM-G, NA-P, GH, CM-M, AD, and LM-H. Obtained and processed lung autopsy specimens: CS-L, CS-G, and CL. Conducted cytokine determinations: L-JA, AC-L, GR-M, and EM-G. Conducted histological and immunohistochemistry analyses: JC-P, CS-L, and CS-G. Performed statistical analyses of the data: JC-P, EC-P, YB-M, and MM-S. Provided reagents: LJ-A, TR-R, CS-L, JG, LM-H, LP-B, GD-C, CC-G, JS-H, PS-D, JR, FA-M, EG-L, CH-C, SAK, AZ, and JZ. Discussed the manuscript: JC-P, TR-R, FA-M, SAK, AZ, and JZ. Drafted the manuscript: JC-P, SAK, AZ, and JZ. All authors contributed to the article and approved the submitted version.

## Funding

JC-P was supported by the National Council of Science and Technology of Mexico (CONACyT) to achieve his PhD degree (CONACyT-CVU 737347). The current study received funding from institutional research grants of INER, from the UNAM-INER interinstitutional collaboration agreement (UNAM: 43355-3065-17-XI-15, to FA-M), from the Dirección General de Personal Académico de la Universidad Nacional Autónoma de México (UNAM, grant #IV201020 to ES), and from CONACyT under the research contracts: CONACYT-Support for scientific research, technological development and innovation in health during COVID-19 contingency (CONACyT-COVID-19), with the project numbers 313517 and 00311999 to TR-R; SECTEI/050/2020, Secretaría de Ciencia, Tecnología e Innovación de la Ciudad de México (SECTEI CDMX); FORDECYT/10SE/2020/05/14-06 and FORDECYT/10SE/2020/05/14-07 from the Fondo Institucional de Fomento Regional para el Desarrollo Científico y Tecnológico y de Innovación (FORDECYT) to JZ.

## Conflict of Interest

The authors declare that the research was conducted in the absence of any commercial or financial relationships that could be construed as a potential conflict of interest.

The reviewer (MC) has declared a shared affiliation, with no collaboration with the author (SK), to the handling editor, at the time of review.
