# Supplementary material for: Clinical and Immunological Factors That Distinguish COVID-19 From Pandemic Influenza A(H1N1)
Source: Front Immunol. 2021 Apr 21;12:593595. doi: 10.3389/fimmu.2021.593595 (PMC8115405; doi:10.3389/fimmu.2021.593595)
Supplement: Supplementary file 1 [file Table_1.docx]

# Supplementary Tables

| Supplemental Table 1. Primer and probe sequences for the detection of SARS-CoV-2 by RT-PCR. | | | |
| --- | --- | --- | --- |
| Assay/use | **Oligonucleotide** | **Sequence ^a^** | **Concentration ^b^** |
| RdRP gene | RdRp_SARSr-F | GTGARATGGTCATGTGTGGCGG | 600 nM per reaction |
|  | RdRp_SARSr-P2 | FAM-CAGGTGGAACCTCATCAGGAGATGC-BBQ | Specific for 2019-nCoV, will not detect SARS-CoV. 100 nM per reaction mixes with P1 |
|  | RdRP_SARSr-P1 | FAM-CCAGGTGGWACRTCATCMGGTGATGC-BBQ | Pan Sarbeco-Probe will detect 2019-nCoV, SARS-CoV, and bat-SARS-related CoVs.  100 nM per reaction mixed with P2 |
|  | RdRp_SARSr-R | CARATGTTAAASACACTATTAGCATA | 800 nM per reaction |
| E gene | E_Sarbeco_F | ACAGGTACGTTAATAGTTAATAGCGT | 400 nM per reaction |
|  | E_Sarbeco_P1 | FAM-ACACTAGCCATCCTTACTGCGCTTCG-BBQ | 200 nM per reaction |
|  | E_Sarbeco_R | ATATTGCAGCAGTACGCACACA | 400 nM per reaction |
| N gene | N_Sarbeco_F | CACATTGGCACCCGCAATC | 600 nM per reaction |
|  | N_Sarbeco_P | FAM-ACTTCCTCAAGGAACAACATTGCCA-BBQ | 200 nM per reaction |
|  | N_Sarbeco_R | GAGGAACGAGAAGAGGCTTG | 800 nM per reaction |
| ^a^W is A/T; R is G/A; M is A/C; S is G/C. FAM: 6-carboxyfluorescein; BBQ: blackberry quencher. ^b^Optimized concentrations are given in nanomole per liter (nM) based on the final reaction mix, e.g., 1.5µL of a 10µM primer stock solution per 25µL total reaction volume yields a final concentration of 600 nM as indicated in the table. | | | |

| Supplemental Table 2. Clinical symptoms with diagnostic value to differentiate pandemic influenza A(H1N1) and COVID-19. | | | |
| --- | --- | --- | --- |
| Symptom | **OR** | **95% CI** | ***p* value** |
| Influenza | | | |
| Fever | 6.462 | 1.062 to 124.8 | 0.0900 |
| Sore throat | 2.667 | 0.7808 to 9.597 | 0.1204 |
| Rhinorrhea | 4.667 | 1.429 to 16.67 | 0.0129 |
| Thoracic pain | 7.333 | 0.9932 to 149.3 | 0.0847 |
| COVID-19 | | | |
| Dry cough | 5.688 | 1.817 to 19.44 | 0.0038 |
| Diarrhea | 2.593 | 0.5551 to 18.66 | 0.2648 |
| Nausea | 1.724 | 0.3344 to 12.87 | 0.5385 |
| Vomit | 4.500 | 0.6954 to 88.35 | 0.1785 |
| Odds ratio (OR) and 95% confidence intervals (CI) for pandemic influenza A(H1N1) or COVID-19 were estimated by bivariate logistic regression analysis. | | | |

| Supplemental Table 3. Clinical characteristics and immune factors with diagnostic value to differentiate critically ill pandemic influenza A(H1N1) and COVID-19 patients. | | | |
| --- | --- | --- | --- |
| Characteristic | **OR** | **95% CI** | ***p* value** |
| ALP | 0.982 | 0.9665 to 0.9955 | 0.0202 |
| LDH | 0.993 | 0.9894 to 0.9975 | 0.0030 |
| Procalcitonin | 0.568 | 0.2854 to 0.9037 | 0.0440 |
| SOFA | 0.648 | 0.4495 to 0.8515 | 0.0065 |
| IL-1β | 29.08 | 5.699 to 403.4 | 0.0013 |
| IL-1RA | 0.998 | 0.9965 to 0.9994 | 0.0151 |
| IL-2 | 0.806 | 0.6829 to 0.9132 | 0.0031 |
| IL-4 | 1.788 | 1.141 to 3.154 | 0.0254 |
| IL-5 | 1.471 | 1.194 to 1.95 | 0.0018 |
| IL-7 | 0.940 | 0.8790 to 0.9957 | 0.0492 |
| IL-12(p70) | 1.299 | 1.136 to 1.609 | 0.0025 |
| IL-13 | 1.689 | 1.291 to 2.401 | 0.0008 |
| IL-17A | 1.168 | 1.026 to 1.387 | 0.0411 |
| TNF-α | 0.958 | 0.9226 to 0.9830 | 0.0072 |
| CCL3 | 0.825 | 0.6783 to 0.9472 | 0.0220 |
| CCL11 | 1.025 | 1.011 to 1.044 | 0.0021 |
| G-CSF | 0.963 | 0.9396 to 0.9892 | 0.0004 |
| VEGF | 1.023 | 1.009 to 1.045 | 0.0092 |
| Odds ratio (OR) and 95% confidence intervals (CI) for COVID-19 were estimated by bivariate logistic regression analysis. | | | |

| Supplemental Table 4. Clinical and immune factors that differentiate pandemic influenza A(H1N1) subjects from the combined cohort of moderate and severe COVID-19 patients. | | | |
| --- | --- | --- | --- |
| Characteristic | **OR** | **95% CI** | ***p* value** |
| ALP | 0.978 | 0.9620 to 0.9915 | 0.0041 |
| LDH | 0.992 | 0.9884 to 0.9962 | 0.0002 |
| Procalcitonin | 0.501 | 0.2448 to 0.8122 | 0.0181 |
| PaO2/FiO2 | 1.012 | 1.003 to 1.023 | 0.0217 |
| AKIN | 0.278 | 0.07901 to 0.9091 | 0.0380 |
| Coinfection | 0.267 | 0.0844 to 0.7991 | 0.0207 |
| Antibiotics | 0.761 | 0.5659 to 0.9938 | 0.0542 |
| SOFA | 0.610 | 0..4284 to 0.7849 | 0.0010 |
| APACHE II | 0.892 | 0.7922 to 0.9834 | 0.0350 |
| IL-1β | 7.804 | 2.802 to 32.79 | 0.0010 |
| IL-1RA | 0.997 | 0.9961 to 0.9991 | 0.0045 |
| IL-2 | 0.780 | 0.6602 to 0.8856 | 0.0008 |
| IL-5 | 1.348 | 1.116 to 1.744 | 0.0074 |
| IL-7 | 0.921 | 0.8613 to 0.9747 | 0.0086 |
| IL-12(p70) | 1.246 | 1.108 to 1.49 | 0.0031 |
| IL-13 | 1.5 | 1.191 to 2.008 | 0.0021 |
| IL-15 | 0.954 | 0.8985 to 0.9965 | 0.0914 |
| IFN-γ | 1.282 | 1.1040 to 1.8120 | 0.0251 |
| TNF-α | 0.953 | 0.9180 to 0.9788 | 0.0032 |
| CCL5 | 0.996 | 0.9938 to 0.9988 | 0.0103 |
| CCL11 | 1.024 | 1.01 to 1.042 | 0.0024 |
| G-CSF | 0.96 | 0.9374 to 0.9777 | 0.0001 |
| VEGF | 1.018 | 1.005 to 1.038 | 0.0244 |
| Odds ratio (OR) and 95% confidence intervals (CI) for COVID-19 were estimated by bivariate logistic regression analysis. | | | |

| Supplemental Table 5. Clinical and immune factors associated with severity of disease and mortality in COVID-19 patients. | | | |
| --- | --- | --- | --- |
| Characteristic | **OR** | **95% CI** | ***p* value** |
| Severe disease | | | |
| Illness onset-hospital admission (days) | 1.288 | 1.056 to 1.748 | 0.0433 |
| WBC | 1.845 | 1.273 to 3.331 | 0.0099 |
| Neutrophils | 1.410 | 1.092 to 2.046 | 0.0267 |
| LDH | 1.019 | 1.009 to 1.036 | 0.0042 |
| Antibiotics | 2.778 | 1.316 to 8.295 | 0.0270 |
| SOFA | 5.085 | 1.928 to 39.52 | 0.0252 |
| IL-4 | 2.627 | 1.247 to 6.717 | 0.0225 |
| IL-6 | 1.071 | 1.011 to 1.191 | 0.0881 |
| IL-7 | 1.176 | 1.03 to 1.417 | 0.0424 |
| IL-8 | 1.111 | 1.024 to 1.256 | 0.0427 |
| IL-12(p70) | 1.115 | 1.027 to 1.256 | 0.0300 |
| IL-15 | 1.171 | 1.023 to 1.404 | 0.0452 |
| VEGF | 1.03 | 1.008 to 1.067 | 0.0392 |
| Mortality | | | |
| Prone position | 6.095 | 1.334 to 34.92 | 0.0265 |
| WBC | 1.238 | 1.043 to 1.532 | 0.0264 |
| SOFA | 1.375 | 1.050 to 1.913 | 0.0333 |
| Odds ratio (OR) and 95% confidence intervals (CI) for severe disease and mortality were estimated by bivariate logistic regression analysis. Severe disease is defined as the need for mechanical ventilation and admission to the intensive care unit (ICU). | | | |

## Supplementary Figures


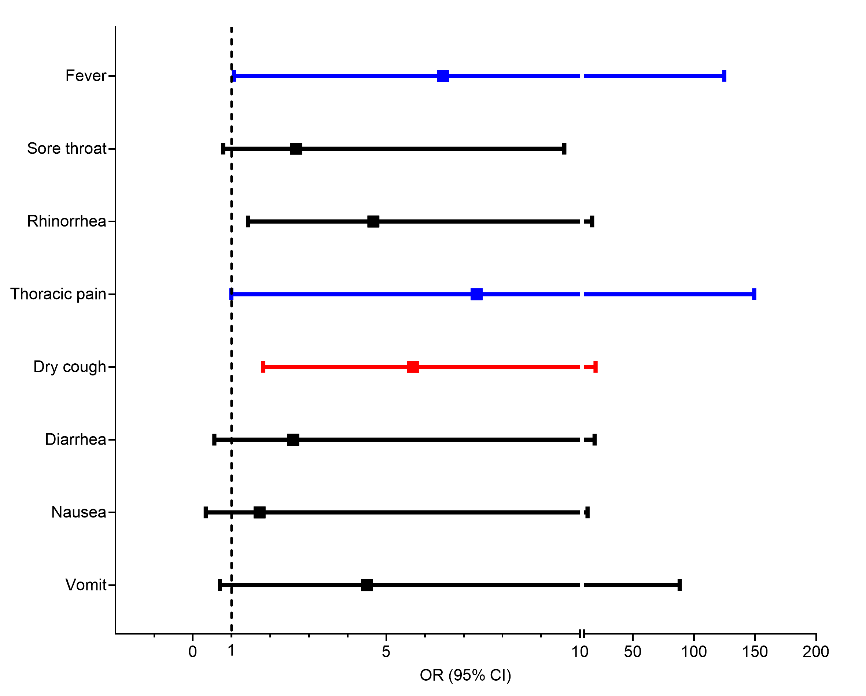


**Supplemental Figure 1. Clinical symptoms with diagnostic value to distinguishing between pandemic influenza A(H1N1) and COVID-19.** Bivariate logistic regression analysis of the symptoms associated with the causative pathogen in patients with acute respiratory distress syndrome (ARDS). The forest plots show the odds ratio (OR) and 95% CI interval values that were non-significant (black) and significant for COVID-19 (red). OR values of factors inversely associated with COVID-19 that instead predict pandemic influenza A(H1N1) are shown in blue color. Absolute OR values are also presented in Supplemental Table 2.


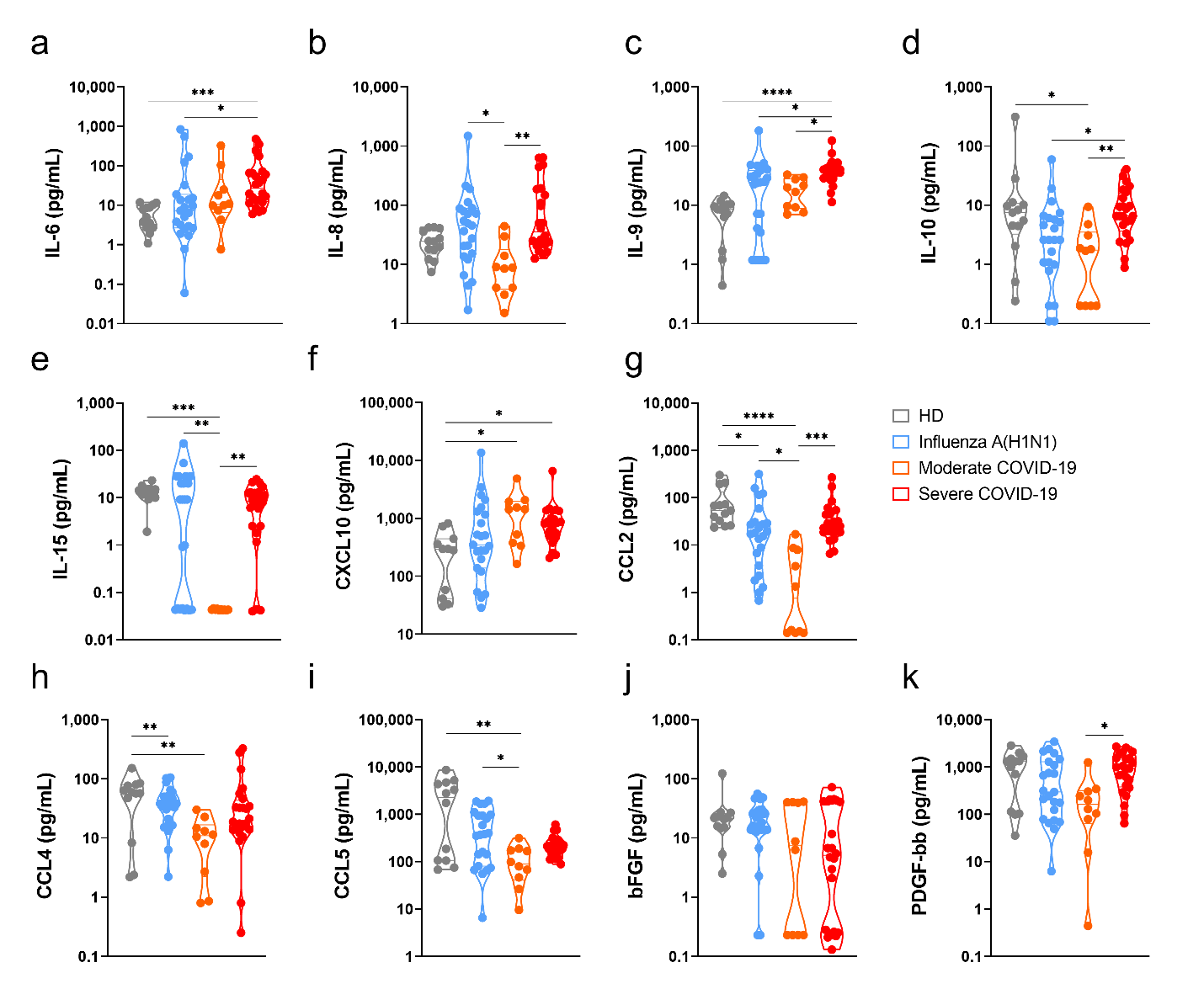
**Supplemental Figure 2. Serum cytokine levels in patients with pandemic influenza A(H1N1) and COVID-19.** Serum levels of cytokines, chemokines, and growth factors in patients with COVID-19 (n=10 moderate, 24 severe) and influenza (n=23), as well as in samples from healthy volunteer donors (HD, n=13), were assessed by Luminex assay. Violin plots show medians and interquartile ranges (IQR). Differences between groups we estimated using the Kruskal-Wallis test with post hoc Dunn´s test. Significant differences are denoted by bars and asterisks: *p≤0.05, **p≤0.01, ***p≤0.001, ****p≤0.0001. (a) IL-6, interleukin 6; (b) IL-8, interleukin 8; (c) IL-9, interleukin 9; (d) IL-10, interleukin 10; (e) IL-15, interleukin 15; (f) CXCL10, C-X-C motif chemokine ligand 10, (g) CCL2, C-C motif chemokine ligand 2; (h) CCL4, C-C motif chemokine ligand 4; (i) CCL5, C-C motif chemokine ligand 5; (j) bFGF, basic fibroblast growth factor; (k) PDGF-BB, platelet-derived growth factor bb.


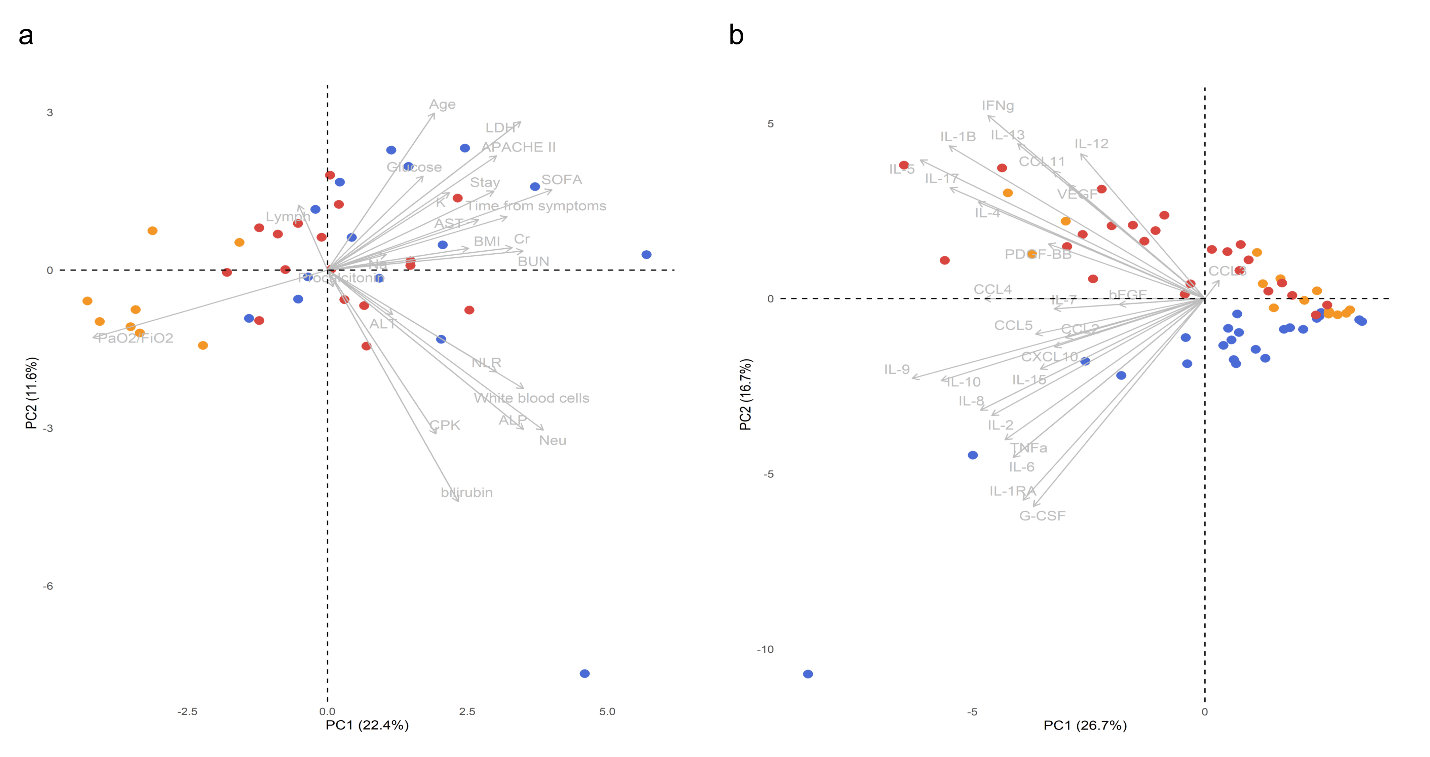
**Supplemental Figure 3.** Principal component analysis (PCA) of the (a) clinical and (b) immunological characteristics of study participants. Each dot represents a single individual, and each color represents a group of participants: blue for severe pandemic influenza A(H1N1), orange for moderate COVID-19, and red for severe COVID-19.


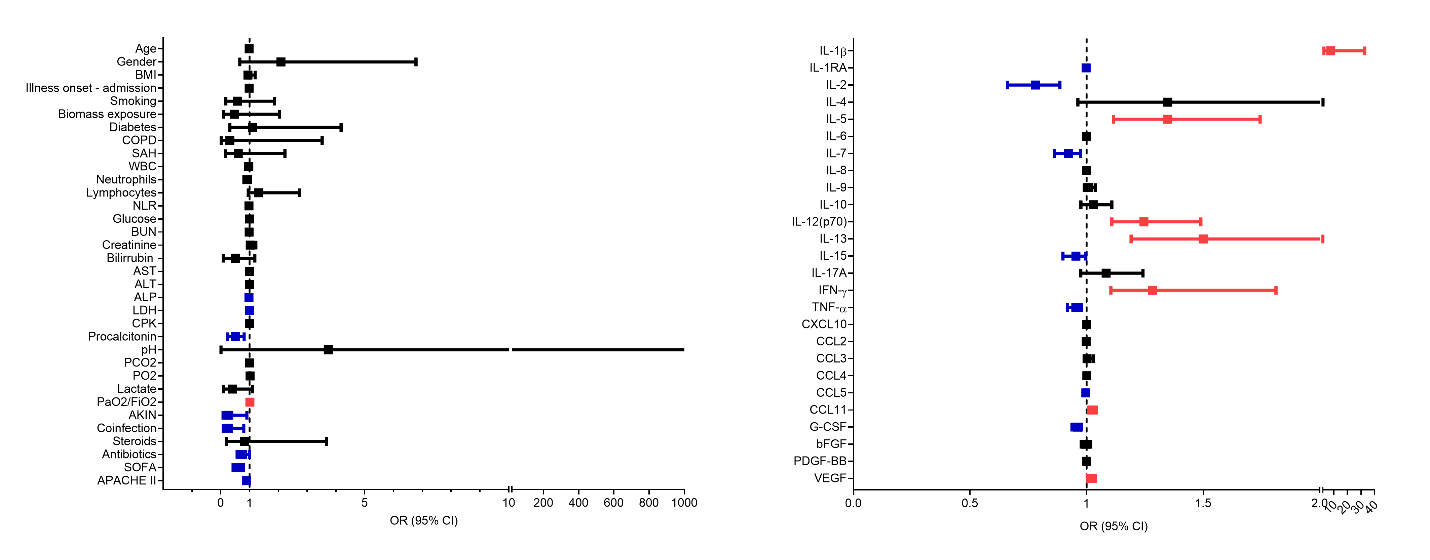
**Supplemental Figure 4. Clinical and immune factors that differentiate pandemic influenza A(H1N1) subjects from patients with moderate and severe COVID-19.** Bivariate logistic regression analysis of the clinical and immunological characteristics associated with COVID-19 and pandemic influenza A(H1N1). The forest plots show the odds ratio (OR) and 95% CI interval values that were non-significant (black) and significant for COVID-19 (red). OR values of factors inversely associated with COVID-19 that instead predict pandemic influenza A(H1N1) are shown in blue color. Absolute OR values are also presented in Supplemental Table 4.


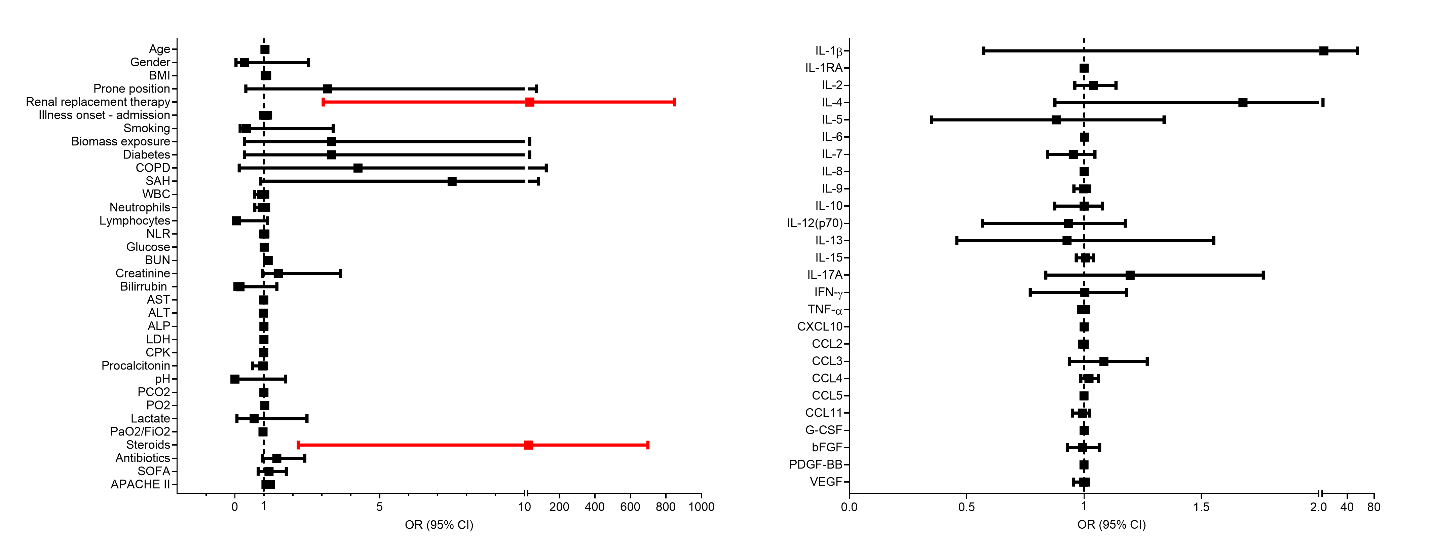
 **Supplemental Figure 5. Clinical and immunological factors associated with mortality in patients with severe pandemic influenza A(H1N1).** Bivariate logistic regression analysis of the clinical and immunological characteristics associated with mortality in patients with influenza A(H1N1) pdm09 virus infection admitted to the intensive care unit (ICU). The forest plots show the odds ratio (OR) and 95% CI interval values. OR values that did not include the null value in the 95% CI were considered significant for mortality and are shown in red color.


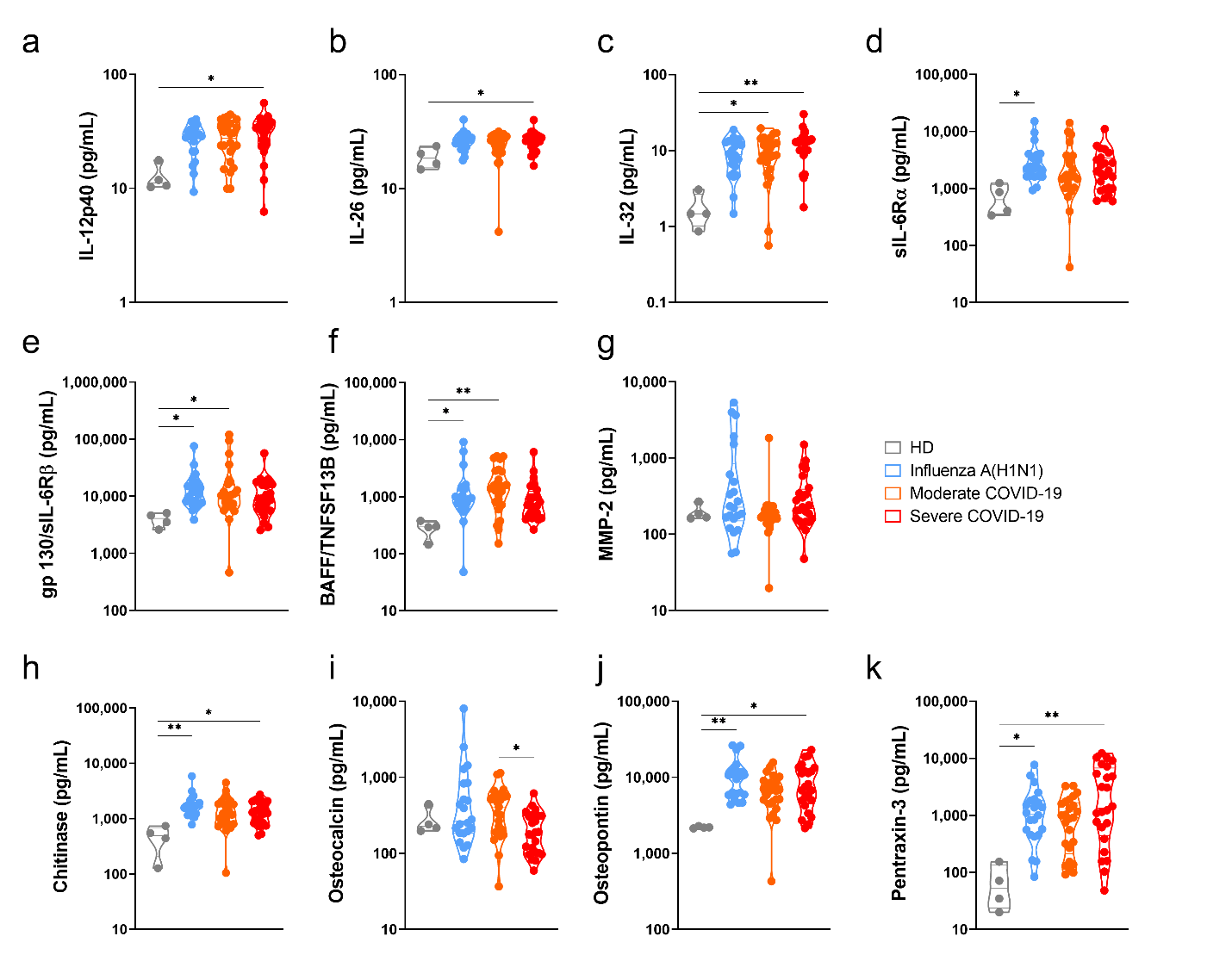
 **Supplemental Figure 6. Other relevant immune mediators in the plasma of patients with pandemic influenza A(H1N1) and COVID-19.** Levels of different soluble immune mediators in plasma samples from healthy volunteer donors (HD, n=4), patients with COVID-19 (n=25 moderate, 24 severe), and pandemic influenza A(H1N1) subjects (n=23), were assessed by Luminex assay. Violin plots display medians and interquartile ranges (IQR). Differences between groups we estimated using the Kruskal-Wallis test with post hoc Dunn´s test. Significant differences are denoted by bars and asterisks: *p≤0.05, **p≤0.01, ***p≤0.001, ****p≤0.0001. (a) IL-12 (p40), interleukin 12 p40 subunit; (b) IL-26, interleukin 26; (c) IL-32, interleukin 32; (d) sIL-6Rα, soluble IL-6 receptor alpha; (e) gp130/sIL-6Rβ, glycoprotein of 130 kDa/soluble IL-6 receptor beta; (f) BAFF/TNFSF13B, B-cell activating factor/tumor necrosis factor ligand superfamily member 13B; (g) MMP-2, metalloprotease 2; (h) chitinase 3/like1; (i) osteocalcin; (j) ostepontin; (k) pentraxin-3.


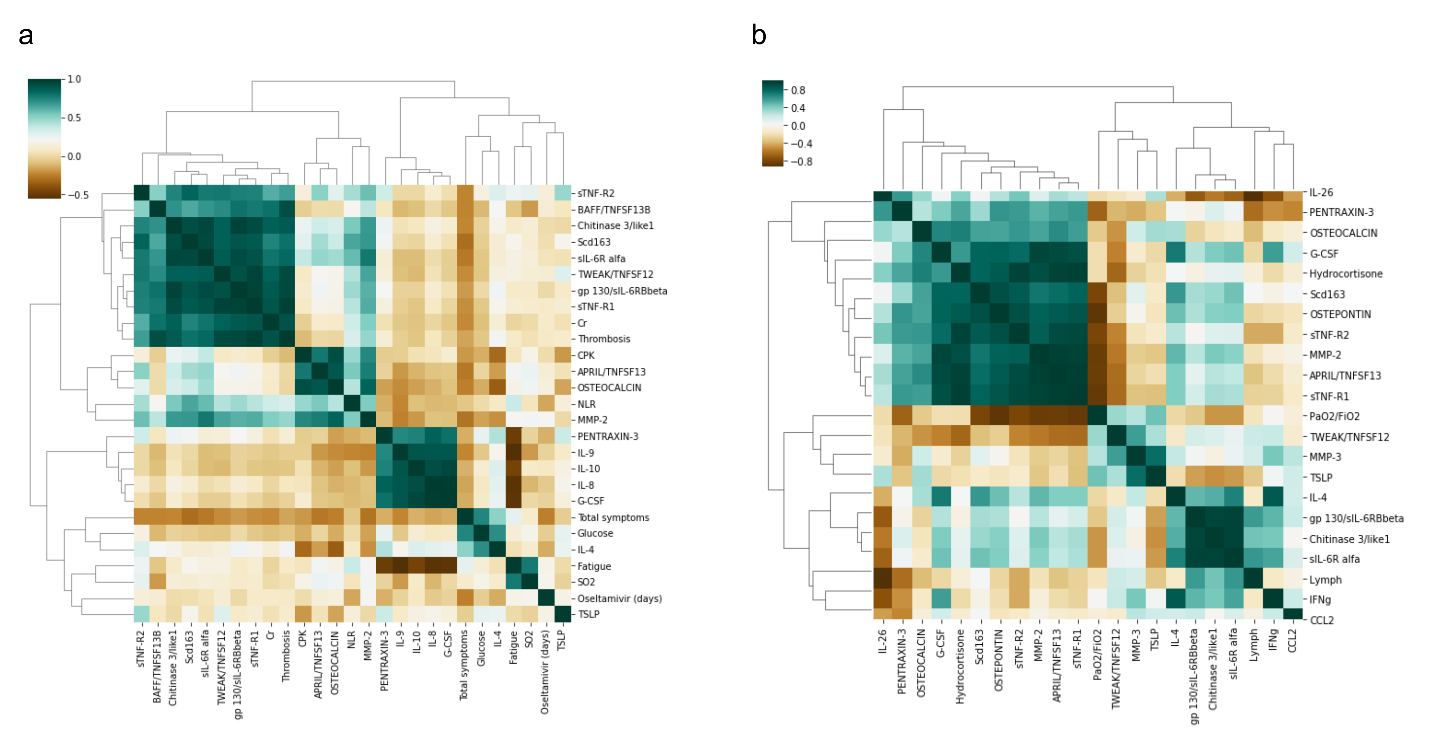
**Supplemental Figure 7.** Multiple correlation analyses of clinical and immunological variables of patients with (a) pandemic influenza A(H1N1) and (b) COVID-19. The heat color map gradients were constructed using correlation coefficient (R) values obtained by the Spearman test for multiple linear correlations. The heat color maps display only those correlations with an R-value ≥ 0.7 or ≥ -0.7.
